# Balancing H^*^ Adsorption/Desorption by Localized 4f Orbital Electrons of Lanthanide Dopants in Carbon‐Encapsulated MoP for Boosted Hydrogen Evolution

**DOI:** 10.1002/advs.202417583

**Published:** 2025-05-14

**Authors:** Jiancheng Li, Juanli Zhao, Yanning Zhang, Yuchen Liu, Maoyuan Li, Riyue Ge, Wenxian Li, Bin Liu

**Affiliations:** ^1^ School of Materials Science and Engineering Shanghai University Shanghai 200444 China; ^2^ Key Laboratory for Optoelectronics and Communication of Jiangxi Province Jiangxi Science & Technology Normal University Nanchang 330038 China; ^3^ College of Science Nanjing Agricultural University Nanjing 210095 China; ^4^ Institute of Coating Technology for Hydrogen Gas Turbines Liaoning Academy of Materials Shenyang 110004 China; ^5^ Institute of Energy Materials Science University of Shanghai for Science and Technology 516 Jungong Road Shanghai 200093 China; ^6^ School of Chemical Engineering University of New South Wales Sydney New South Wales 2052 Australia; ^7^ Australia Research Council Centre of Excellence for Carbon Science and Innovation UNSW Sydney New South Wales 2052 Australia

**Keywords:** binding energy, orbital coupling, rare earth, water electrolysis

## Abstract

MoP is one of the most efficient catalysts for hydrogen evolution reaction (HER). However, molybdenum ion exhibits a strong adsorption ability for H^*^ due to the abundant states in the conduction bands associated with the dispersed nature of Mo 4d electrons, which is difficult to change through interactions with other transition metal dopants of d orbital electrons. Herein, lanthanide (Ln: La, Ce, Sm, Gd, and Yb) dopants of localized 4f orbital are used to hybridize with d and p orbitals of MoP to balance the H^*^ adsorption energy to significantly enhance the HER activity. Density functional theory calculations indicate that the localized 4f orbital of Ln extended 4d orbital electronic density states of Mo to modulate the electron configuration near the Fermi level. Among various Ln‐doped MoP with carbon‐encapsulated catalysts (Ln‐MoP@C) catalysts, Gd‐MoP@C containing Mo 4d, C 2p, P 3p, and Gd 4f and 5d orbitals form significant states at the Fermi level, leading to the high intrinsic HER activity with low overpotentials of 74 and 134 mV at 10 mA cm^−2^ in alkaline and acid electrolytes, respectively. This study provides a guiding principle for selecting dopants to tune electronic structures and enhance the intrinsic catalytic activities of transition metal catalysts through f‐d‐p orbital coupling.

## Graphical abstract

The Pt‐like electronic structure and fast electron transfer channels induced by Ln dopant (Ln: La, Ce, Sm, Gd, and Yb) demonstrate enhanced contribution for hydrogen evolution reaction.

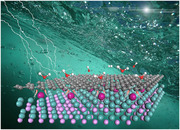

## 1 Introduction

Electrochemical water splitting to produce hydrogen via the cathode hydrogen evolution reaction (HER) is considered a clean, and renewable energy strategy to solve the energy crisis and environmental pollution.^[^ ^1^ ^]^ However, developing efficient catalysts for HER is still challenging due to its sluggish reaction kinetics and elevated overpotential. Currently, platinum (Pt)‐based catalysts remain the preferred choice for HER for their superior catalytic performance. The special 5d^9^6s^1^ valence electron arrangement in Pt allows it to exhibit ideal hydrogen binding energy, lowering the energy barrier and facilitating the rate‐limiting step. However, the practical application of Pt for hydrogen production is hindered by its scarcity and poor durability.^[^ ^2^ ^]^ Therefore, cost‐effective and sustainable alternatives with Pt‐like electronic structures are highly desirable for HER to break through the bottleneck for hydrogen production.

Molybdenum phosphide (MoP) has a hexagonal crystal structure with a molybdenum layer encapsulated by phosphorus atoms with a similar electronic structure (4d^5^5s^1^) as that of Pt for Mo.^[^ ^3^ ^]^ The density functional theory (DFT) calculations reveal that MoP is prone to the adsorption of H because of its plenty of bonding states in conductive bands, and the binding energy decreases toward zero with an increase of H coverage.^[^ ^4^ ^]^ Thus, fine‐tuning the electronic structure of MoP, for example through heteroatom‐doping,^[^ ^5^ ^]^ is expected to improve the intrinsic activity of HER. Indeed, the charge coupling between Fe and Mo in Fe‐doped MoP was demonstrated to effectively tune the d‐band center of the Mo site due to the contribution of electrons in the partially filled d‐valence orbitals of Fe, thus optimizing the free adsorption energy of the H intermediate.^[^ ^6^ ^]^ This is because the d orbital is located at the outermost of transition metals, which are susceptible to external influences of crystal field and coordination environment.^[^ ^7^ ^]^

Owing to the large atomic radius of lanthanide elements to induce abundant lattice distortion and unsaturated coordination sites in the electrocatalysts, the rare‐earth elements, especially the 15 lanthanide elements, have garnered increasing attention as dopants for effectively tuning the electronic properties of catalysts through local charge rearrangement to promote the adsorption of reactants,^[^ ^8^ ^]^ and hence improved catalytic activities.^[^ ^8^, ^9^ ^]^ For instance, the introduction of Yb/La into MoP results in charge transfer to reduce the adsorption‐free energy of hydrogen.^[^ ^8f^ ^]^ It should be noted that the main focus is still on the surface adsorption energy optimization for crucial reaction intermediates. Meanwhile, although the orbital hybridization induced by the unique orbital structure of lanthanides with 4f^n−3^5d^1^6s^2^ or 4f^n−2^6s^2^ outer electronic configurations has attracted more attention to discuss in recent articles.^[^ ^10^ ^]^ the unique contributions resulting from the different electron‐filling states of the 4f orbitals in different rare earth elements have not been discussed in detail.

The hybridization of d orbital electrons of transition metal dopants with MoP can only generate dispersive density of states (DOS) with limited modification of the electronic structure and H^*^ adsorption/desorption ability of MoP. Lanthanides are characterized by their weak shielding effects of 5d6s electrons on 4f electrons, which provides chances for localized 4f electrons to hybridize with 4d electrons in MoP. Herein, we selected five different lanthanides (Ln: La, Ce, Sm, Gd, and Yb) to rationally construct Ln‐doped MoP with carbon‐encapsulated catalysts (Ln‐MoP@C) to explore the possibility of balancing the H^*^ adsorption/desorption ability on Ln‐MoP@C via the hybridization of 4f and 5d orbitals of Ln with Mo 4d, P 3p, and C 2p. The DOS revealed that, depending on the electron configurations, electrons in Ln 4f and 5d orbitals made different contributions to modify the electronic structure around the active sites. Benefiting from the orbital hybridization between the Ln atoms and the surrounding atoms, the density states of Mo 4d‐P 3p‐C 2p orbitals formed a Pt‐like cusp feature at the Fermi level in Gd‐MoP@C. Meanwhile, Ln atoms act as electron donors to affect the surrounding electron segregation in the Ln‐MoP@C, which increases the electron density of Mo and C to accelerate the adsorption and desorption of hydrogen and molecular water, respectively.^[^ ^11^ ^]^ The higher electron‐donating ability of Gd compared to other Ln atoms makes the Gd site act as a strong Lewis acid site to promote the desorption of intermediates during the HER process. Motivated by DFT prediction, we developed a facile method to synthesize Ln‐MoP@C via a phosphating Mo‐dopamine precursor flower spheres with trapped Ln ions. It was found that the introduction of Ln in MoP@C significantly affects the charge distribution around the doped sites at the atomic level, influencing the HER activity in both alkaline and acid electrolytes. Consequently, the as‐prepared Gd‐MoP@C exhibited the highest HER performance with a low overpotential of 74 and 134 mV at 10 mA cm^−2^ in alkaline and acid electrolytes, respectively.

## 2 Results and Discussion

### 2.1 DFT Screening of Ln‐MoP@C Electronic Structure

**Figure 1:**
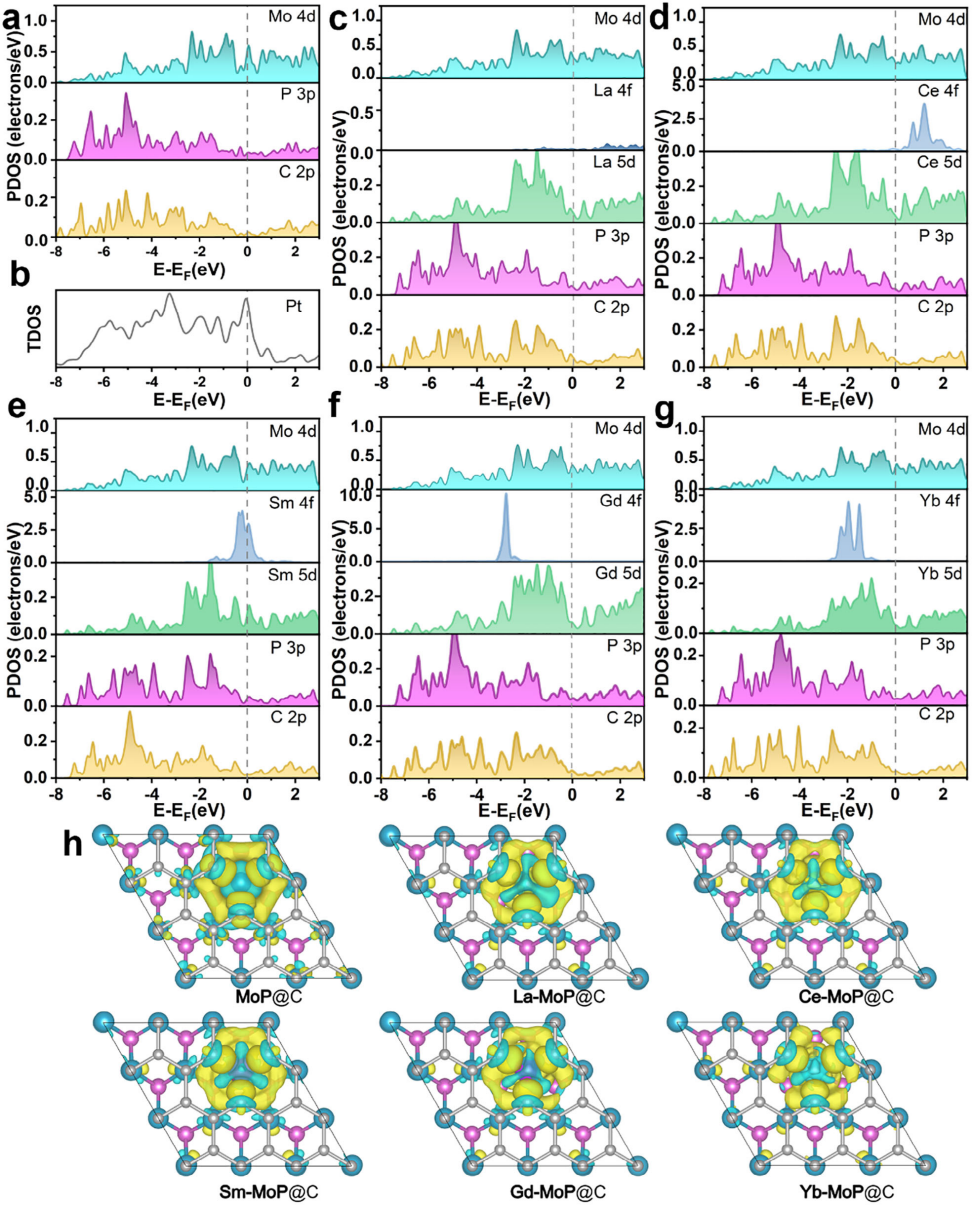
Theoretical insights of Ln‐MoP@C and electronic structure regulation. a) the PDOS of MoP@C. b) TDOS of Pt. c–g) the PDOS of La‐MoP@C, Ce‐MoP@C, Sm‐MoP@C, Gd‐MoP@C, and Yb‐MoP@C, respectively. h) The differential charge density diagrams of MoP@C and Ln‐MoP@C.

The Ln‐doped MoP with C layer wrapping (Ln‐MoP@C, Ln: La, Ce, Sm, Gd, and Yb) models are first profiled by DFT calculations (Figure S1, Supporting Information). Here, the standard Ln pseudopotentials (i.e., with valence 4f electrons) have been chosen for the calculation process of Ln‐MoP@C, so as to take into account the unique 4f orbitals. The electronic wavefunctions and energy levels of La, Ce, and Gd are described as 4f^n−3^5d^1^6s^2^ configuration (n is the total number of 4f, 5d, and 6s electrons), while for Sm and Yb, 4f^n−2^6s^2^ is used. The obtained PDOS (projected density of state) of MoP@C, La‐MoP@C, Ce‐MoP@C, Sm‐MoP@C, Gd‐MoP@C, and Yb‐MoP@C are shown in **Figures** **1**a,c–g and S2 (Supporting Information). In the case of La, as shown in Figure 1c, a negligible signal is exhibited in the 4f orbital, which is consistent with the fact that no 4f electrons in its 4f orbital. The relatively unstable 4f orbital filling in Ce and Sm atoms makes their electrons distribute on both sides of the Fermi level and easily be excited above the Fermi level (Figure 1d,e). The 4f orbitals of Gd and Yb are filled in half‐full and fully‐filled states, respectively, which makes their state density mainly concentrated below the Fermi level as shown in Figure 1f,g.

Although the lanthanide 4f orbital is localized, the hybridization between the 4f orbital and the delocalized 5d6s orbitals changes the local electronic structure. From the viewpoint of orbital chemistry,^[^ ^12^ ^]^ a lower 4f^n−2^6s^2^ transition energy would make an easier 4f‐5d6s orbital self‐hybridization on the Ln atoms, which would distort the local electronic structure through the bonding interaction between the Ln atoms and the surrounding atoms.^[^ ^8c^ ^]^ As shown in Figure 1a, the P 3p‐Mo 4d‐C 2p orbital hybridization mainly occurs far away from the Fermi level (≈−5 eV) for MoP@C. However, for Gd‐MoP@C, the obvious self‐hybridization between 4f and 5d orbitals of Gd has existed at −3 eV, which leads to the extension of the 5d orbital electronic density states to a high energy level (Figure 1f). As a result, the Gd 5d and P 3p have the peak overlaps at −2.9/−1.5/−0.5 eV, while those between Gd 5d and C 2p are observed at −2.9/−0.5 eV. It should be noticed that the Gd doping also indirectly influences the Mo related bonding near the Fermi level. Extra overlapping peaks are observed at −0.83/0 eV for Mo 4d and P 3p, and 0 eV for Mo 4d and C 2p. These electronic redistributions around the Gd site are also reflected by the change of differential charge diagram before and after Gd doping as shown in Figure 1h and Figure S3 (Supporting Information). More importantly, above mentioned electronic structure modification forms an electronic density state peaks’ overlapping at the Fermi level, which includes Gd 5d, P 3p, Mo 4d and C 2p orbitals, being similar to that of Pt as shown in Figure 1b and Figure S2 (Supporting Information).^[^ ^13^ ^]^ Such Pt‐like characteristic means that the electron state at the Fermi level can balance the bonding and debonding ability of these electrons, which may maximize the electrochemical property improvement of Gd‐MoP@C. However, in the cases of La‐MoP@C, Ce‐MoP@C, Sm‐MoP@C, and Yb‐MoP@C, Ln dopant induces electronic structure modification also occurs, but without the Pt‐like characteristics at the Fermi level.

In order to clarify the charge redistribution after Ln doping, the average Bader charge of MoP@C and Ln‐MoP@C are compared in Table S1 (Supporting Information). The Bader charge of Mo and C in Ln‐MoP@C are more positive than those in MoP@C. It indicates that the electron‐donating ability of Mo in Ln‐MoP@C is weakened compared with MoP@C and the increased charge in C mainly comes from the contribution of Ln as an electron donor. Meanwhile, the increased electronic density of states around C will also facilitate H adsorption on the catalyst surface, benefiting the HER process. Among them, Gd‐MoP@C exhibits the highest positive average Bader charge of C (0.22). In an alkaline electrolyte, electron‐deficient Gd and electron‐abundant C can act as Lewis acid and base sites, respectively. Their large electrostatic potential difference can enhance the polarization of H_2_O and the kinetics of H─OH bond breaking.^[^ ^14^ ^]^ Based on the above electronic structure analysis, Gd dopant in MoP@C is expected to bring a more balanced adsorption/desorption of intermediates for electrocatalytic improvement.

### 2.2 Synthesis and Structural Characterization

**Figure 2:**
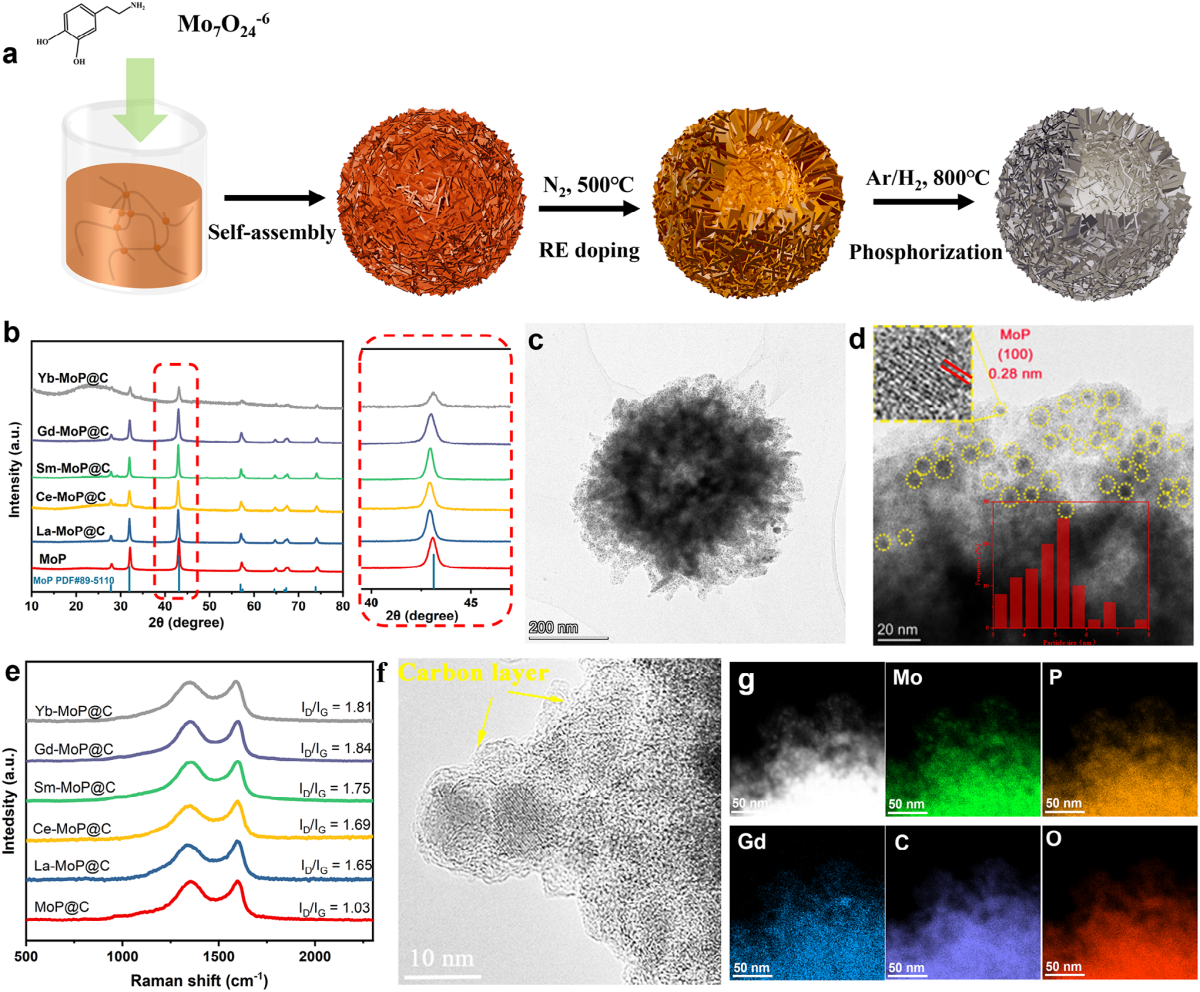
Composition and structure characterization of Gd‐MoP@C. a) schematic of the fabrication process of Ln‐MoP@C. b) XRD patterns of MoP@C, La‐MoP@C, Ce‐MoP@C, Sm‐MoP@C, Gd‐MoP@C, and Yb‐MoP@C. c) TEM image, d,f) HRTEM images of Gd‐MoP@C. e) Raman spectra of Ln‐MoP@C. g) EDX mapping of the Gd‐MoP@C.

A series of 3D self‐assembled Ln‐MoP@C are prepared to validate our discovery. As presented in **Figure** **2**a, the Ln‐doped MoP is performed using an impregnation technique to adsorb Ln ions on the precursor surface and followed by a high‐temperature phosphating treatment, which induces Ln into MoP. First, a hierarchical Mo–DPA hybrid (DP‐400) assembled by ultrathin 2D nanosheets is fabricated by self‐assembly of Mo_7_O_24_^6−^ and dopamine in a weakly alkaline environment. A large number of ultrathin 2D nanosheets eventually form a nanoflower‐structured DP‐400 with ≈300 nm particle size as shown in a scanning electron microscopy (SEM) image (Figure S4, Supporting Information). After that, Ln atoms are introduced into DP‐400 by adopting rare earth nitrates as the Ln sources. Then, the obtained mixture is calcined in a tubular furnace at 500 °C to cleave the self‐assembled structure between dopamine and Mo_7_O_24_^6−^, and convert to carbon‐stabilized Ln decorated molybdenum hybrid (Ln‐Mo@C). As shown in Figure S5 (Supporting Information), a hollow structured Gd‐Mo@C with a flaky raised surface can be obtained by introducing the Gd atoms and following the pyrolysis process. Meanwhile, the X‐ray diffraction (XRD) pattern of Gd‐Mo@C indicates that no obvious phase structure is formed during the initial annealing process (Figure S6, Supporting Information). The broad peak between 20° and 30° can be attributed to the carbon formed after the pyrolysis of dopamine. Finally, the Ln atoms doped MoP stabilized by carbon are fabricated by converted Ln‐MoP@C at 800 °C with sodium hypophosphite as a phosphorus source. During the phosphating process, a small amount of P may combine with the carbon matrix. However, the porous nature of the carbon matrix, combined with the highly oxidizable property of P, makes it exist in the carbon matrix as an oxide of phosphorus.^[^ ^15^ ^]^ This greatly restricts the exposure of P in the carbon matrix as potential actives, but P can still enhance the electrical conductivity and wettability of the carbon matrix.^[^ ^16^ ^]^

The XRD patterns of the fabricated Ln‐MoP@C electrocatalysts are shown in Figure 2b. The main peak position (≈43°) of Ln‐MoP@C, which fits the (1 0 1) planes of MoP, slightly shifts to lower 2θ values compared to the bare sample, which can be attributed to the lattice stretching of MoP caused by Ln (0.98–1.16 Å) doping with a larger ion radius than Mo (0.66 Å).

The SEM image (Figure S7, Supporting Information) and low‐magnification TEM image (Figure 2c) of Gd‐MoP@C show that the as‐prepared materials have a uniform hollow structure with nano‐sheets on the surface. The STEM image and corresponding elemental maps suggest a homogeneous distribution of Mo, Gd, P, C, and O elements in Gd‐MoP@C as shown in Figure 2g, indicating the well‐dispersed Gd dopant. Measured from the High‐resolution TEM (HRTEM) image of Gd‐MoP@C, the interplanar distances of lattice fringes is 0.27 nm, which is assigned to the (1 0 0) lattice plane of MoP (Figure 2d). The size of MoP nanoparticles is ≈4–5 nm, in which MoP avoids agglomerating into large particles due to the protective effect of the carbon layers.^[^ ^17^ ^]^ Considering the requirement of catalysis, the open 3D nanoflower structure benefits from sufficient exposure of the active sites. Meanwhile, the nanosheets composed in such a structure can pump liquid‐phase electrolytes onto the catalytic surface because of the strong capillary forces.^[^ ^18^ ^]^ As a result, it can reduce the adhesion at the gas‐solid interface and accelerate the desorption of hydrogen bubbles from the electrocatalyst surface.

**Figure 3:**
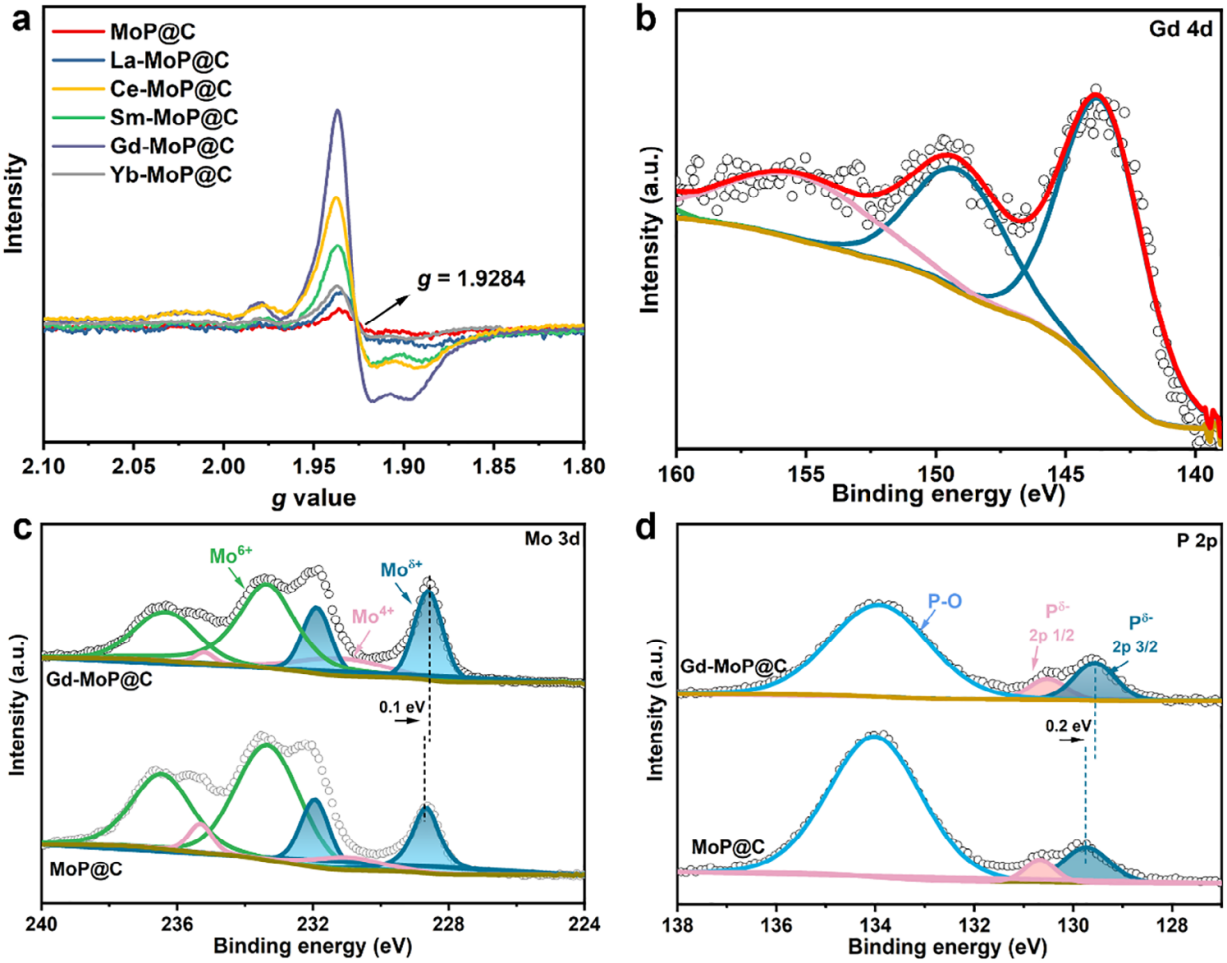
Electronic structure of as‐prepared electrocatalysts. a) Electron paramagnetic resonance of MoP@C, La‐MoP@C, Ce‐MoP@C, Sm‐MoP@C, Gd‐MoP@C, Yb‐MoP@C. high‐resolution XPS spectra of b) Gd 4d for Gd‐MoP@C, and c) Mo 3d, d) P 2p for Gd‐MoP@C and MoP@C.

As shown in Figure 2e, the Raman spectrum of the as‐prepared catalysts all show two typical peaks of D‐band (≈1345 cm ^−1^) and G‐band (≈1858 cm ^−1^), correlated to the E_2g_ mode of *sp* ^2^ carbon, indicating the existence of carbon in the prepared catalysts. The comparison of bright‐field and high angle annular dark‐field scanning transmission electron microscopy images (Figure 2f) uncovers typical carbon‐encapsulated MoP nanoparticles, which can also be obtained from the lattice fringes with a distance of ≈0.36 nm in graphitic carbon (Figure S8, Supporting Information). The carbon layers on the surface of the catalyst can promote electron transport during the reaction and effectively reduce the activity decay of the catalyst during HER. Meanwhile, based on the spectrum in Figure 2e, the I_D_/I_G_ values of MoP@C, La‐MoP@C, Ce‐MoP@C, Sm‐MoP@C, Gd‐MoP@C, and Yb‐MoP@C are calculated to be 1.03, 1.65, 1.69, 1.75, 1.84, and 1.81, respectively, reflecting the increase of carbon defect after Ln doping. Since the carbon defects act as anchoring sites, its increased concentration contributes to the high dispersion of MoP nanoparticles in Ln‐MoP@C. Moreover, the specific defect structure also plays an important role in the local electronic structure of MoP@C and Ln‐MoP@C. A characteristic peak at *g* values of 1.9284 appears in the measured Electron paramagnetic resonance in **Figure** **3**a, indicating the unpaired electron spin in unsaturated Mo atoms caused by Ln doping.^[^ ^19^ ^]^ The stronger intensity of the characteristic peak of Gd‐MoP@C than that of the MoP@C indicates the increasing of unsaturated Mo atoms with the Gd doping, which allows further regulation of the electronic structure and provides more electrons to participate in the catalytic reaction.

The elemental compositions and valence states of each element on the surface of MoP@C and Gd‐MoP@C (Figure S9, Supporting Information) are unveiled using X‐ray photoelectron spectroscopy (XPS). The high‐resolution Gd 4d XPS spectrum of Gd‐MoP@C in Figure 3b, which matches with the Gd^3+^,^[^ ^20^ ^]^ confirms that Gd atoms have been successfully incorporated into the MoP lattice. So do the Ce and Sm as indicated by the Ce 3d and Sm 3d spectra of Ce‐MoP@C and Sm‐MoP@C in Figure S10 (Supporting Information).^[^ ^21^ ^]^ The high‐valence state of Ce^3+^/Ce^4+^ and Sm^3+^ also indirectly indicates that it primarily exists in the form of doping.^[^ ^22^ ^]^ Figure 3c displays the Mo 3d spectrum of MoP@C and Gd‐MoP@C. The Mo 3d of the Gd‐MoP@C curve can be divided into three couples at 228.3/231.9 eV, 230.9/235.1 eV, and 233.5/237.0 eV, which are ascribed to Mo^δ+^ (0 <δ <4), Mo^4+^ and Mo^6+^, respectively.^[^ ^23^ ^]^ The Mo^4+^ and Mo^6+^ are associated with the Mo─O bonds in MoO_2_ and MoO_3_, respectively, which is formed by the oxidation of Mo atoms on the surface of MoP in air to stabilize the unpaired electrons in Mo orbitals.^[^ ^3a^ ^]^ The Mo^δ+^ peaks with lower binding energy belong to the Mo─P bond, which are typical peaks in MoP. These Mo^δ+^ peaks in Gd‐MoP@C negatively shift to lower binding energy compared to the Mo^δ+^ peaks in MoP@C, revealing that the electron cloud moves to Mo after the Gd doped. As shown in Figure 3d, the high‐resolution P 2p spectrum shows two peaks at ≈129.8 and ≈130.6 eV, which correspond to P 2p_1/2_ and P 2p_3/2_ of P^δ−^ in P─Mo bonds of MoP, respectively. The peak at ≈134.1 eV is ascribed to oxidized phosphorus. Interestingly, the P^δ−^ peak in Gd‐MoP@C also shows a negative shift compared to that in MoP@C like the Mo^δ+^ peak. As uncovered by the simulation results in the last section, Gd works as an electron donator in Gd‐MoP@C. Therefore, the gathered electrons around Mo and P should come from Gd, rather than grabbing from each other. The decrease in the electron density of Gd can adjust the electronic state to facilitate the adsorption of H_2_O and the kinetics of H─OH bond breaking as Lewis acid sites.^30^ In the C 1s spectrum (Figure S11, Supporting Information), the peaks located at 284.8, 285.5, and 288.1 eV are assigned to C═C/C─C, C─O─P, and C─O, respectively. After the Gd doping, the peak area ratio of C═C/C─C bond decreases, indicating that the C defect increases, which is consistent with the Raman results.

### 2.3 Electrochemical Performance

**Figure 4:**
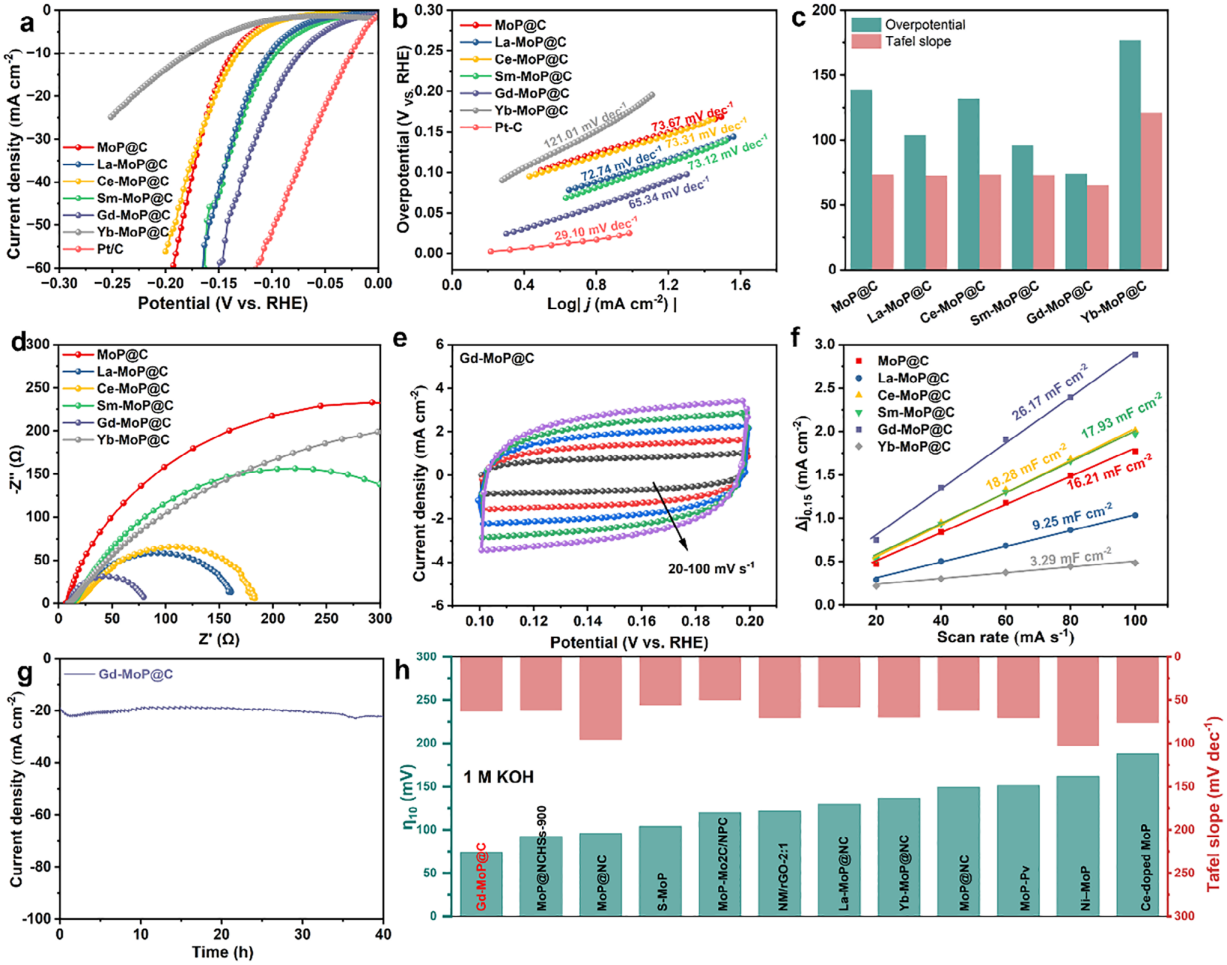
HER performances of different samples in 1 m KOH. a) Polarization curves and b) Tafel plots of Pt/C, MoP@C, La‐MoP@C, Ce‐MoP@C, Sm‐MoP@C, Gd‐MoP@C, and Yb‐MoP@C in 1 m KOH. c) The comparison of Ln‐MoP@C on overpotential and Tafel slope. d) Nyquist plots recorded at an overpotential of 100 mV for MoP@C, La‐MoP@C, Ce‐MoP@C, Sm‐MoP@C, Gd‐MoP@C, and Yb‐MoP@C in 1 m KOH. e) ECSA and f) C_dl_ of MoP@C, La‐MoP@C, Ce‐MoP@C, Sm‐MoP@C, Gd‐MoP@C, and Yb‐MoP@C in 1 m KOH. g) The stability test of Gd‐MoP@C in 1 m KOH. h) Overpotential at a current density of 10 mA cm^−2^ and Tafel slope for other electrocatalysts in recent years.

The electrochemical HER activities of Ln‐MoP@C are measured via a standard three‐electrode cell in N_2_‐saturated 1 m KOH aqueous at first, together with those of commercial Pt/C and MoP@C for references. **Figure** **4**a displays the polarization curves (linear sweep voltammetry, LSV) of all as‐prepared catalysts in 1.0 m KOH medium at a scan rate of 5 mV s^−1^. In order to explore the intrinsic behavior of the electrocatalysts, IR compensation is applied in the electrochemical measurements. It is observed that all Ln‐MoP@C electrodes exhibit superior HER activity to bare MoP@C (139 mV at 10 mA cm^−2^) electrodes. Interestingly, the Gd‐MoP@C displays the best activity among Ln‐MoP@C electrodes owing to its specific electron state peak at the Fermi level. It has a low overpotential of 74 mV at 10 mA cm^−2^ in 1.0 m KOH medium, which is smaller than those of La‐MoP@C (104 mV), Ce‐MoP@C (132 mV), Sm‐MoP@C (96 mV), Yb‐MoP@C (177 mV) and MoP@C (139 mV). Moreover, although the activity of Gd‐MoP@C is inferior to the commercial Pt/C, its overpotential at 10 mA cm^−2^ is lower compared to reported MoP‐based electrocatalysts in literature (Figure 4h).

The Tafel slope for HER which is obtained by fitting the Tafel equationη = a + blog(j), provides insight into the catalytic kinetics and intrinsic properties of Ln‐MoP@C electrodes. Figure 4b presents the Tafel slopes of Pt/C, MoP@C, La‐MoP@C, Ce‐MoP@C, Sm‐MoP@C, Gd‐MoP@C and Yb‐MoP@C. The Gd‐MoP@C shows a smaller Tafel slope of 65.34 mV dec^−1^ than MoP@C (73.67 mV dec^−1^), La‐MoP@C (72.74 mV dec^−1^), Ce‐MoP@C (73.31 mV dec^−1^), Sm‐MoP@C (73.12 mV dec^−1^), and Yb‐MoP@C (121.01 mV dec^−1^). Notably, the value of Gd‐MoP@C is closer to the Tafel slope of Pt/C compared to recently focused MoP catalysis materials (like MoP‐P_v_ and Ni‐MoP in Figure 4h). Moreover, these Tafel slope values also indicate that the HER kinetics of prepared catalysts follow a Volmer–Heyrovsky mechanism, where the rate‐determining step is the Heyrovsky step.^[^ ^24^ ^]^ The exchange current density (*j*_0_) is a kinetic‐related parameter that can reflect the efficiency of charge transfer between the electrode and the catalyst surface.^[^ ^25^ ^]^ As shown in Table S2 (Supporting Information), the *j*_0_ of Gd‐MoP@C is 3.02 mA cm^−2^, which is better than MoP@C (1.63 mA cm^−2^), La‐MoP@C (1.76 mA cm^−2^), Ce‐MoP@C (1.24 mA cm^−2^), Sm‐MoP@C (1.99 mA cm^−2^) and Yb‐MoP@C (1.01 mA cm^−2^). The larger *j*_0_ in the micro polarization region ensures that polarization reactions of Gd‐MoP@C require lower resistance.

The electrical conductivity of prepared catalysts in alkaline media, which is evaluated by electrochemical impedance spectroscopy (EIS), assesses the charge transfer kinetics at the interface of the obtained catalysts. As illustrated in Figure 4d, Gd‐MoP@C displays the smallest value of charge transfer resistance (*R*_ct_) (69 Ω) than other Ln‐MoP@C and bare MoP@C, indicating rapid electron transfer efficiency. The low *R*_ct_ should be attributed to the modified local electronic environments induced by Gd doping, which accelerates charge transfer. At the same time, their electrochemical double‐layer capacitance (*C*_dl_) is estimated and compared in Figure S12 (Supporting Information) and Figure 4e, so as to characterize the electrochemically active surface area (ECSA). Specially, as shown in Figure 4f, the *C*_dl_ value of Gd‐MoP@C (26.17 mF cm^−2^) is larger than MoP@C and other Ln‐MoP@C, suggesting that it exhibits larger ECSA to expose the highest effective active area during the catalysis process. Moreover, the good stability of Gd‐MoP@C can be recognized from Figure 4g. After a 40 h test with a current density of 20 mA cm^−2^, the Gd‐MoP@C electrode still maintains high catalytic performance. The SEM image and XRD pattern of Gd‐MoP@C in Figure S13 (Supporting Information) reveal that the Gd‐MoP@C keeps excellent structural and composition stability after this test. Further, the long‐term stability of the electrocatalyst in high current density of 100 and 500 mA cm^−2^ was additionally conducted, and was found to be stable for 150 h (Figure S14, Supporting Information).The overall water splitting measurement was carried out using a two‐electrode system with Gd‐MoP@C as the cathode and commercial RuO_2_ as the anode. Bubbles of generated H_2_ and O_2_ are clearly observed from the experiment setup, as displayed in the picture of Figure S15 (Supporting Information). The Gd‐MoP@C||RuO_2_ electrode catalyzed water electrolysis takes only 1.6 V at a current density of 10 mA cm^−2^.

**Figure 5:**
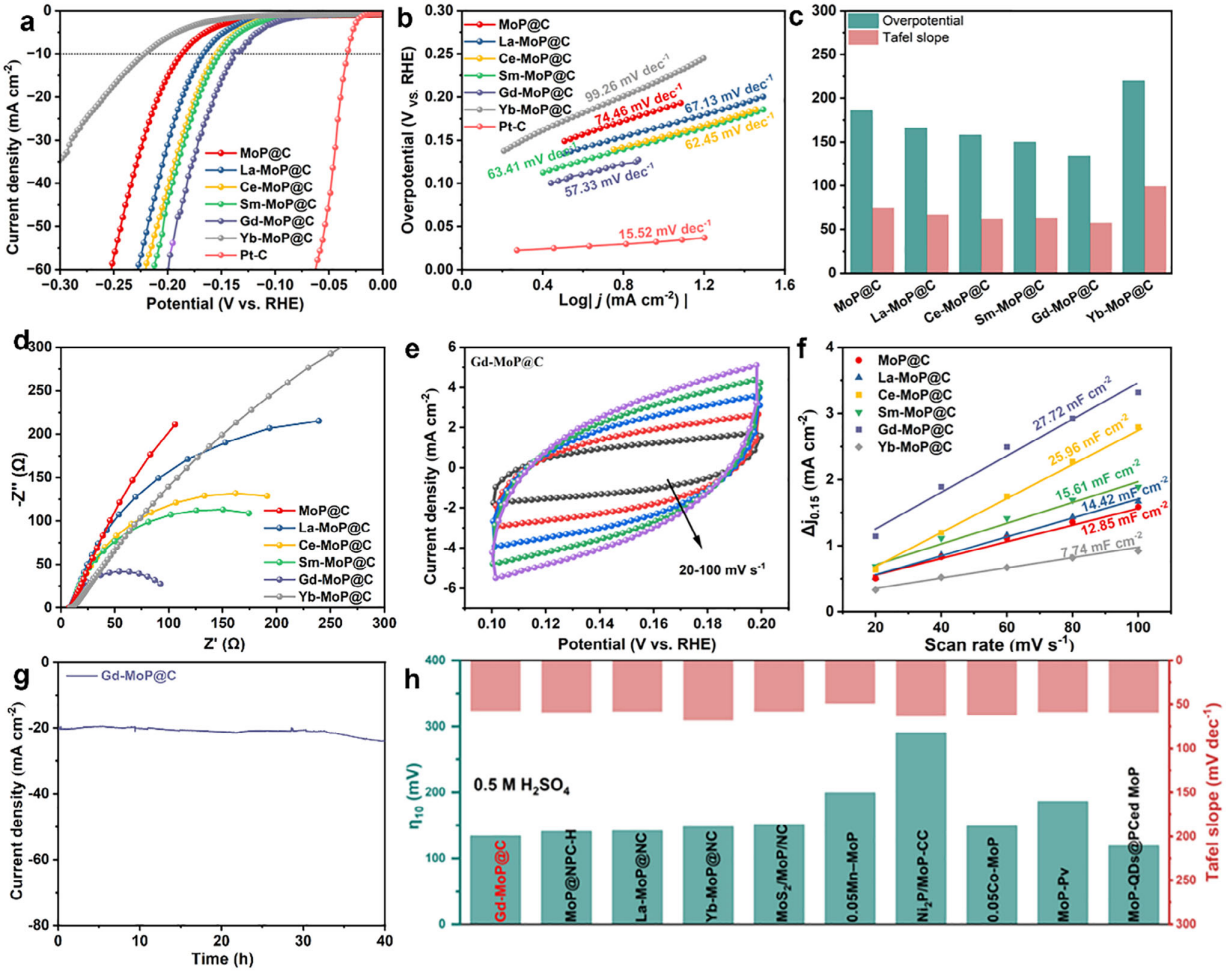
HER performances of different samples in 0.5 _M_ H_2_SO_4_. a) LSV curves and b) Tafel plots of Pt/C, MoP@C, La‐MoP@C, Ce‐MoP@C, Sm‐MoP@C, Gd‐MoP@C, and Yb‐MoP@C in 0.5 m H_2_SO_4_. c) The comparison of Ln‐MoP@C on overpotential and Tafel slope. d) Nyquist plots recorded at an overpotential of 100 mV for MoP@C, La‐MoP@C, Ce‐MoP@C, Sm‐MoP@C, Gd‐MoP@C, and Yb‐MoP@C in 0.5 m H_2_SO_4_. e) ECSA and f) *C*_dl_ of MoP@C, La‐MoP@C, Ce‐MoP@C, Sm‐MoP@C, Gd‐MoP@C, and Yb‐MoP@C in 0.5 m H_2_SO_4_. g) The stability test of Gd‐MoP@C in 0.5 m H_2_SO_4_. h) Overpotential at a current density of 10 mA cm^−2^ and Tafel slope for other electrocatalysts in recent years.

The HER activities of Ln‐MoP@Cs are also measured in 0.5 m H_2_SO_4_. The Gd‐MoP@C exhibits a small overpotential of 134 mV at the current density of 10 mA cm^−2^ with a Tafel slope of 57.33 mV dec^−1^, which is better than those of bare MoP@C (186 mV and 74.46 mV dec^−1^), La‐MoP@C (166 mV and 67.13 mV dec^−1^), Ce‐MoP@C (158 mV and 62.45 mV dec^−1^), Sm‐MoP@C (150 mV and 63.41 mV dec^−1^), and Yb‐MoP@C (220 mV and 99.26 mV dec^−1^) as shown in **Figure** **5**a–c. The low activity of Yb‐MoP@C may originate from different crystallinity (Figure 1b), which results in a higher impedance to restrict the reaction kinetics and makes it hard to reduce the overpotential.^[^ ^23^ ^]^ Meanwhile, all Ln‐MoP@Cs have relatively close *j*_0_ values (Table S2, Supporting Information) in 0.5 m H_2_SO_4_, which are 1.52, 1.68, 1.67, 1.7, 1.8, 1.63 mA cm^−2^ for MoP@C, La‐MoP@C, Ce‐MoP@C, Sm‐MoP@C, Gd‐MoP@C, Yb‐MoP@C, respectively. The exchange current and the activity of MoP@C and Ln‐MoP@C change in the same trend in 0.5 m H_2_SO_4_. The EIS data in Figure 5d reveal that Gd‐MoP@C exhibits a low *R*_ct_ of 84 Ω in acidic solutions, implying a faster charge transfer process in acidic solutions. This result is consistent with the LSV and Tafel slope results shown in Figure 5c. From Figure 5e,f, and Figure S16 (Supporting Information), the Gd‐MoP@C still maintains the highest *C*_dl_ and ECSA, indicating the abundant exposure of active sites during the HER process in 0.5 m H_2_SO_4_. The ECSA‐normalized LSV curves for HER in alkaline and acid media were calculation in Figure S17 (Supporting Information). From the Figure S17 (Supporting Information), the Gd‐MoP@C catalyst still exhibit the highest activity, suggesting intrinsically improved HER activity on the Gd‐MoP@C. Being similar to the stability test in alkaline, the Gd‐MoP@C used electrode can also maintain stable current density at 20 mA cm^−2^ for 40 h in 0.5 m H_2_SO_4_ as shown in Figure 5g, revealing the good stability of Gd‐MoP@C in both 0.5 m H_2_SO_4_ and 1 m KOH.

### 2.4 Mechanism Investigation

**Figure 6:**
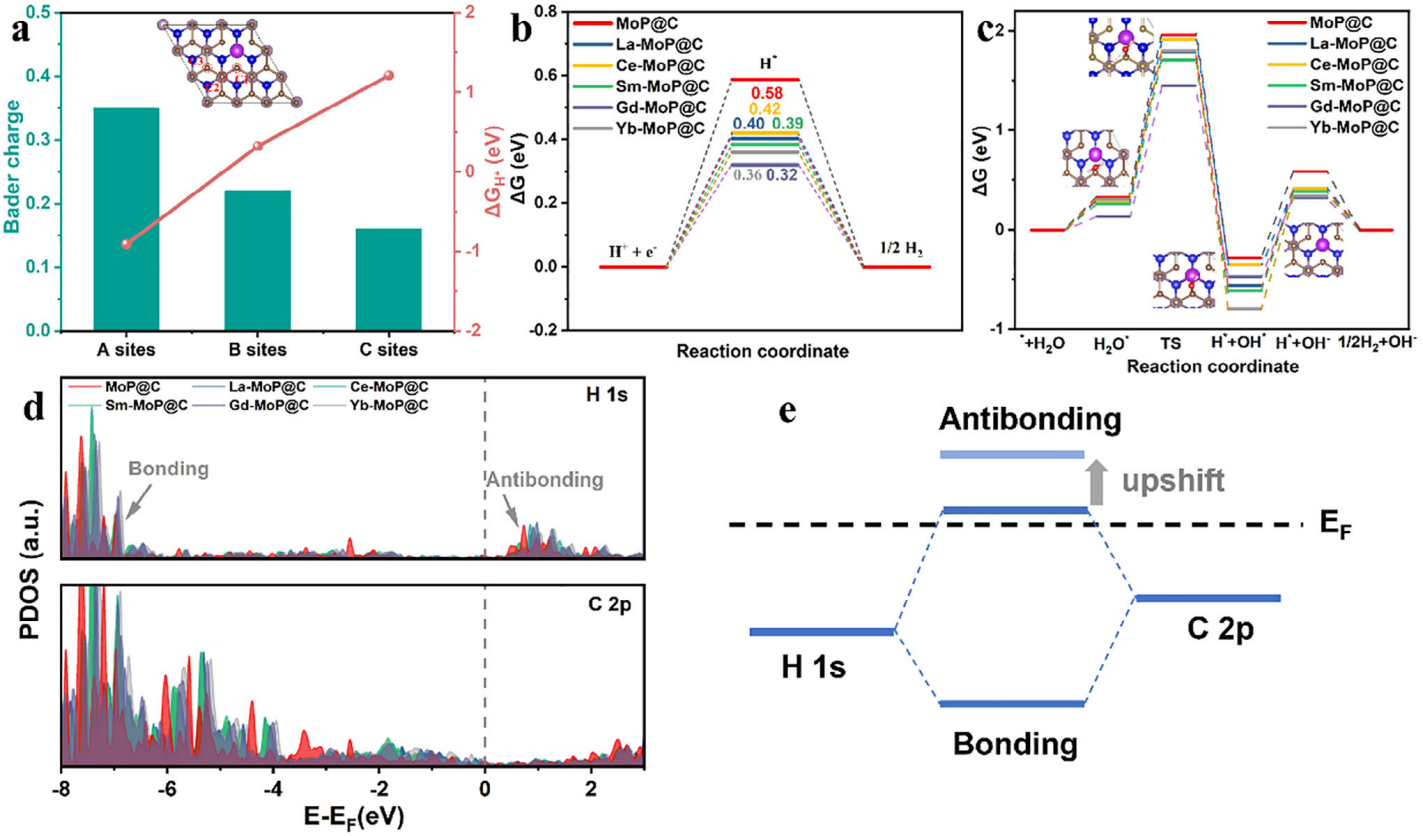
Theoretical investigation. a) Bader charge and Gibbs free energy of different carbon sites in Gd‐MoP@C. Gibbs free energy diagram of HER in b) acid media and c) alkaline media for MoP@C, La‐MoP@C, Ce‐MoP@C, Sm‐MoP@C, Gd‐MoP@C, and Yb‐MoP@C, respectively. d) PDOS of H atoms adsorbed on the surface of MoP@C and Ln‐MoP@C. e) Schematic illustration of the formation of H─C bonds for the Ln‐MoP@C. With the Ln atoms doped in MoP@C, corresponding antibonding states upshift from *E*_F_ to a higher energy.

In order to understand the atomic/molecular‐level catalytic mechanism of the Ln‐MoP@C in both acid and alkaline electrolytes, the adsorption/desorption process of intermediates on the Ln‐MoP@C surface, together with the related free energy change (ΔG) and electronic structure, is explored by DFT simulations. The calculated ΔG_H*_ on the potential adsorption sites of the C layer (C1‐site, C2‐site, and C3‐site), Mo‐site, and Gd‐site, are shown in Figure S18 (Supporting Information). Compared with the C3‐site (1.21 eV) being far away from the vacancy, the Mo‐site (0.65 eV) and C2‐site (0.32 eV) around the vacancy have a decreased adsorption‐free energy, which can accelerate the adsorption of hydrogen. ΔG_H*_ of C1‐site (−0.89 eV) and Gd‐site (−0.91 eV) is too negative, resulting in strong hydrogen adsorption that restricts the desorption process of hydrogen. Interestingly, the hydrogen adsorption barrier for the different C sites also raises with the average Bader charge decrement of the C atoms, further corroborating the reactivity trends (**Figure** **6**a). Therefore, H is more likely to be adsorbed and desorbed at the C2 site during the HER process.

The ΔG_H*_ of Ln‐MoP@C and MoP@C exhibited in Figure 6b are positive for all models, but the doping of Ln elements does reduce ΔG_H*_ toward zero. Among them, the minimum ΔG_H*_ of 0.32 eV is observed for Gd‐MoP@C, indicating that absorbing H on the surface of the catalyst becomes easier after Gd doping than other studied Ln‐MoP@C and MoP@C. To explore the underlying mechanism behind above ΔG_H*_ modification induced by Ln dopant, the PDOS of C 2p and H 1s orbitals for C─H bonds on the adsorbed surface are depicted in Figure 6d. It is observed that the H 1s‐C 2p bonding resonance occurs below the Fermi level, while the antibonding states of H 1s are mainly distributed above the Fermi level. After Ln doping, the energy peak position of the antibonding states changes toward a higher energy direction. Figure 6e shows a schematic illustration of the interaction between H 1s and C 2p orbitals. When the adsorbed H hybridizes with the C 2p orbital on the catalyst surface, the adsorbed states will be divided into localized states and antibonding states. The occupancy degree of the antibonding states directly affects the adsorption process of hydrogen on the carbon sites. For MoP@C, the antibonding states are around the Fermi level, indicating a relatively high occupancy. In the case of Ln‐MoP@C, Ln doping leads to more adsorbate antibonding states being pulled above the Fermi level, corroborating a decreased occupancy rate of the antibonding states and eventually resulting in a stronger C─H interaction (Figure 6e).^[^ ^26^ ^]^

The calculated kinetic energy barrier of Ln‐MoP@C and MoP@C for HER in alkaline media is shown in Figure 6c. H_2_O will preferentially aggregate near the Ln atoms and adsorb on the C1 sites, which can be attributed to the adsorption promotion effect of rare earth atoms as oxyphilic sites on H_2_O. It is found that the H_2_O adsorption energy of the Ln‐MoP@C is reduced compared to MoP@C. The most negative water adsorption energy is observed on the Gd‐MoP@C surface, suggesting that water is easier adsorbed on Gd‐MoP@C than on others. Meanwhile, the energy barrier for H_2_O dissociation on Gd‐MoP@C is the lowest compared to the other Ln‐MoP@Cs and MoP@C. After water splits into H^*^ and OH^*^, the significant free energy decrement will promote the strong adsorption of H^*^ and OH^*^ on the C1 site and C2 site for further desorption, respectively.^[^ ^27^ ^]^ Among these three steps of adsorption of water, H_2_O dissociation, and the desorption of OH^*^, the energy barrier that needs to be overcome in the dissociation process of H_2_O is the largest, indicating the H_2_O dissociation is the rate‐determining step in the alkaline HER kinetics of Ln‐MoP@C catalysts. In all, the Gd doping promotes the water adsorption, water dissociation, and OH^*^ desorption steps, which expedite the HER process of Gd‐MoP@C in an alkaline medium.

The calculated energy barrier for each reaction step proves that the improved intrinsic activity of Ln‐MoP@C compared to MoP@C originates from Ln doping‐induced local electronic structure variation. As well known, although the 4f orbital is localized, the 4f‐5d‐6s hybridization provides extended valence electron states (Figure 1d) and active electron states near the Fermi level for Ln elements. The latter is associated to the fact that the electronegativity of Ln (1.1–1.25) is only nearly half of Mo (2.15).^[^ ^8c^ ^]^ When the Ln is doped into MoP@C, the obvious hybridization peaks among the 5d orbital of Ln, C 2p and P 3p orbitals present (Figure 1), ensuing the Ln dopant stability. Meanwhile, the electronic states near the Fermi level are activated more or less. The most exciting result is observed for Gd‐MoP@C, where Gd 5d, P 3p, Mo 4d and C 2p orbitals bonding peaks locate below but very close to and/or at the Fermi level. Such characteristic makes Gd‐MoP@C own suitable bonding energies of intermediates during HER, namely, providing its best balance of bonding and deboning ability among studied materials. Eventually, Gd‐MoP@C exhibits excellent HER activity in experiments.

## 3 Conclusion

In summary, we modulated the electronic structure of Ln‐MoP@C by the hybridization effect of the Ln multi‐orbital structure and the fine‐tuning of Ln 4f electrons. We revealed that Ln elements act as electron donors to change the electron segregation of surrounding Mo, P, and C atoms, which electron‐deficient Ln and electron‐abundant C could serve as the Lewis acid‐base pairs for intermediates adsorption via upshifting the antibonding states of C─H band. Meanwhile, 4f‐5d6s orbitals hybridization of Ln optimizes electronic structure around the doping sites, enhancing Ln dopant stability and activating the electronic states near the Fermi level. Especially, the Gd‐MoP@C achieved remarkable electrocatalytic activity toward acid/alkaline HER, with a lower ΔG_H*_ and energy barrier for the H_2_O dissociation than those of MoP@C and other studied Ln‐MoP@C. Finally, this study doesn't only offer a series of Ln‐MoP@C catalysts for hydrogen production but also provides a fundamental understanding of the effect of f‐orbital for lanthanide rare earth elements in electron‐dependent activity, which is expected to inspire a prospective field of rare earth dopant modified electrocatalysts and combined with other optimization methods to rational design catalyst for future energy‐related applications.

## 4 Experimental Section

### Fabrication of Mo‐Dopamine Precursor (DP‐400)

In a typical synthesis,^[^ ^28^ ^]^ 250 mg of ammonium molybdate ((NH_4_)_6_Mo_7_O_24_._4_H_2_O) and 400 mg dopamine hydrochloride were dissolved in the 70 mL deionized (DI) water. Subsequently, 150 mL of ethanol was quickly poured into the above solution. After stirring for 10 min, 400 µl of ammonia water was dropped into the mixed solution for further stirring 2 h at room temperature to obtain the claret mix‐solution. Then, the mixture was washed with DI water and ethanol three times, and finally dried in a vacuum drying oven at 60 °C to obtain the final Mo‐dopamine precursor (DP‐400).

### Fabrication of Ln‐MoP@C

200 mg DP‐400 was dispersed in a beaker filled with 10 mL DI water and ultrasounded for 10 min. Then, 0.05 mmol Ln(NO_3_)_3_ (Ln: La, Ce, Sm, Gd, and Yb) was added to the beaker. After continuously stirring at 70 °C to obtain the dry products as Ln‐DP‐400. Then, the Ln‐MoP@C was obtained by heating the Ln‐DP‐400 in a tube furnace under N_2_ atmosphere at 500 °C for 3 h, followed by phosphating at 800 °C for 1 h using sodium hypophosphite as P sources.

### Characterizations

Field emission scanning electron microscope (JSM‐7500F) and field‐emission transmission electron microscope (JEM‐2100F) were used to investigate the morphology and structure. The XRD (smartlab,) with Cu K_α_ radiation was employed to characterize the crystalline phase. XPS was conducted on a Thermo Scientific K‐Alpha, using C 1s (248.8 eV) as a reference. EPR) measurements were recorded on an EMXplus EPR spectrometer.

### Electrochemical Measurements

The electrochemical measurements were performed in a conventional three‐electrode system with an Autolab PGSTAT (Metrohm Autolab). A graphite plate was used as the counter electrode in all measurements. A saturated calomel electrode (SCE) and Ag/AgCl electrode were used as the reference electrodes in 1 m KOH and 0.5 m H_2_SO_4_, respectively. The catalyst ink was prepared by dispersing 4 mg as‐prepared sample into a mixture (700 µL water, 250 µL isopropyl alcohol, and 50 µL 5% Nafion solution) and ultrasonic dispersion for 30 min to obtain a homogeneous ink. Then, was deposited on the glassy‐carbon electrode to obtain a working electrode after the solvent was dried naturally. The electrochemical impedance spectroscopy (EIS) tests were recorded with frequencies from 0.1 to 100000Hz. The *C*_dl_ is estimated via measuring CV curves from 0.1 to 0.2 V (vs RHE) without a redox process at varying rates from 20 to 100 mV s^−1^. *j_0_* and the symmetry factor (α) were obtained by fitting kinetic current density (*j_k_*) at small current density region into the Butler–Volmer equation as follows:

(1) $$j_{k}=j_{0}\left[\exp\left(\frac{\alpha F}{RT}\eta \right)-\exp\left(\frac{-\left(1-\alpha \right)F}{RT}\eta \right)\right]$$

In the Micropolarization region (−10 to 10 mV), Bulter–Volmer equation can be simplified to

(2) $$j_{0}=j\frac{j}{\eta }\frac{RT}{F}$$

where *j* is the measured current density, *η* is the overpotential, *R* is the universal gas constant, *T* is the temperature, and *F* is Faraday's constant.^[^ ^29^ ^]^

### Computation Details

Density functional theory (DFT) calculations were performed by the Vienna ab initio simulation package (VASP),^[^ ^30^ ^]^ which applied the projector augmented wave (PAW) method^[^ ^31^ ^]^ to treat the core‐electron interactions and the generalized gradient approximation (GGA)^[^ ^32^ ^]^ in the form of the Perdew–Burke–Ernzerhof functional for exchange‐correlation potential. To calculate the density of state and electron density difference, 550 eV cutoff energy was assigned to the plane‐wave basis. 10^−5^ eV as the convergence threshold was set for the iteration in selfconsistent‐field (SCF). 0.05 eV Å^−1^ as the maximum force component was set for all atomic positions for geometry optimizations by BFGS algorithm.The Brillouin Zone k‐point was set by a 3 × 3 × 1 Monkhorst–Pack grid. The adsorption energy (*E*_ads_) of species *X* (*X* can be H, OH, or H_2_O) is calculated by

(3) Eads=E(X/slab)−E(X)−E(slab)

The free energies were evaluated by

(4) $$\Delta G_{\mathrm{ads}}=\Delta E_{\mathrm{ads}}+\Delta E_{\mathrm{ZPE}}-T\Delta S_{\mathrm{ads}}$$

where E_ZPE_ is the zero‐point energy, and S is the entropy, respectively. The temperature is considered as 298.15 K.

## Conflict of Interest

The authors declare no conflict of interest.

## Supporting information



Supporting Information

## Data Availability

The data that support the findings of this study are available in the supplementary material of this article.
