# Supplementary material for: Balancing H* Adsorption/Desorption by Localized 4f Orbital Electrons of Lanthanide Dopants in Carbon‐Encapsulated MoP for Boosted Hydrogen Evolution
Source: Adv Sci (Weinh). 2025 May 14;12(23):2417583. doi: 10.1002/advs.202417583 (PMC12199375; doi:10.1002/advs.202417583)
Supplement: Supplementary file 1 — Supporting Information [file ADVS-12-2417583-s001.docx]

Balancing H* Adsorption/Desorption by Localized 4f Orbital Electrons of Lanthanide Dopants in Carbon-Encapsulated MoP for boosted Hydrogen evolution

Jiancheng Li ^a^, Juanli Zhao ^b^, Yanning Zhang ^a^, Yuchen Liu ^c^, Maoyuan Li^a, d^, Riyue Ge ^e^, Wenxian Li ^f, g^* and Bin Liu ^a^*

^a^ *School of Materials Science and Engineering, Shanghai University, Shanghai 200444, China*

^b^ *Key Laboratory for Optoelectronics and Communication of Jiangxi Province, Jiangxi Science & Technology Normal University, Nanchang 330018, China*

^c^ *College of Science, Nanjing Agricultural University, Nanjing, 210095, China*

^d^ *Institute of Coating Technology for Hydrogen Gas Turbines, Liaoning Academy of Materials, Shenyang, 110004, China*

^e^ *School of Fashion & Textiles, The Hong Kong Polytechnic University Hung Hom, Hong Kong, 999077 China*

^f^ *School of Materials Science and Engineering,* *University of New South Wales, Sydney, NSW 2052, Australia*

^g^ *UNSW Materials & Manufacturing Futures Institute, UNSW, Sydney, NSW 2052, Australia*

c
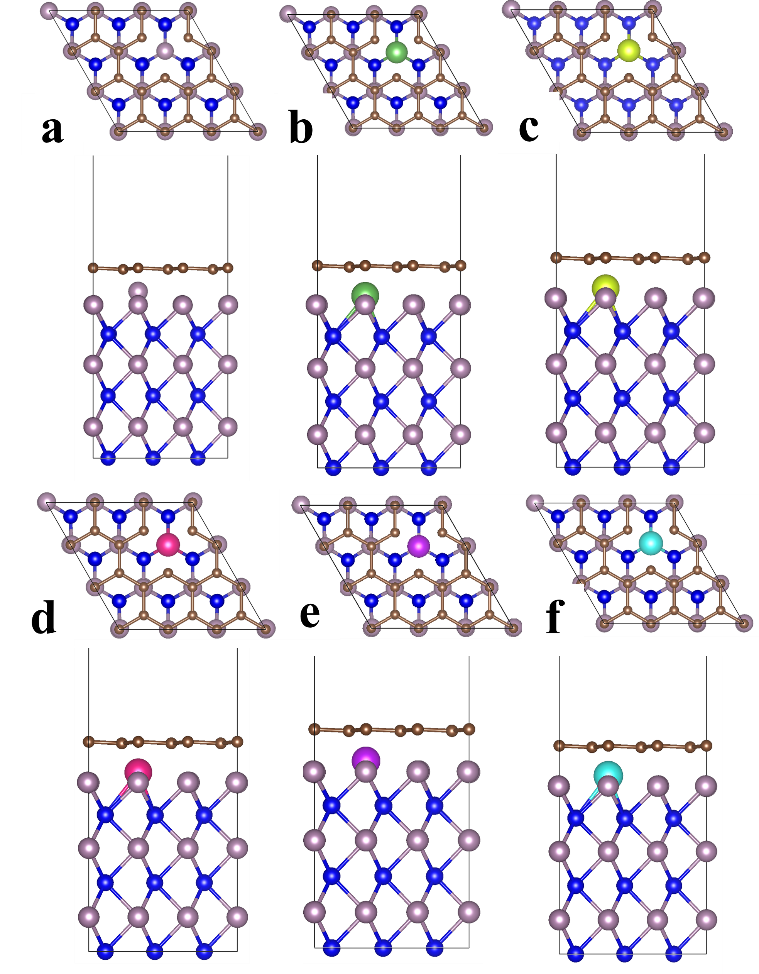


**Figure S1** Calculation models of (a) MoP@C, (b) La-MoP@C, (c) Ce-MoP@C, (d) Sm-MoP@C, and (f) Yb-MoP@C.


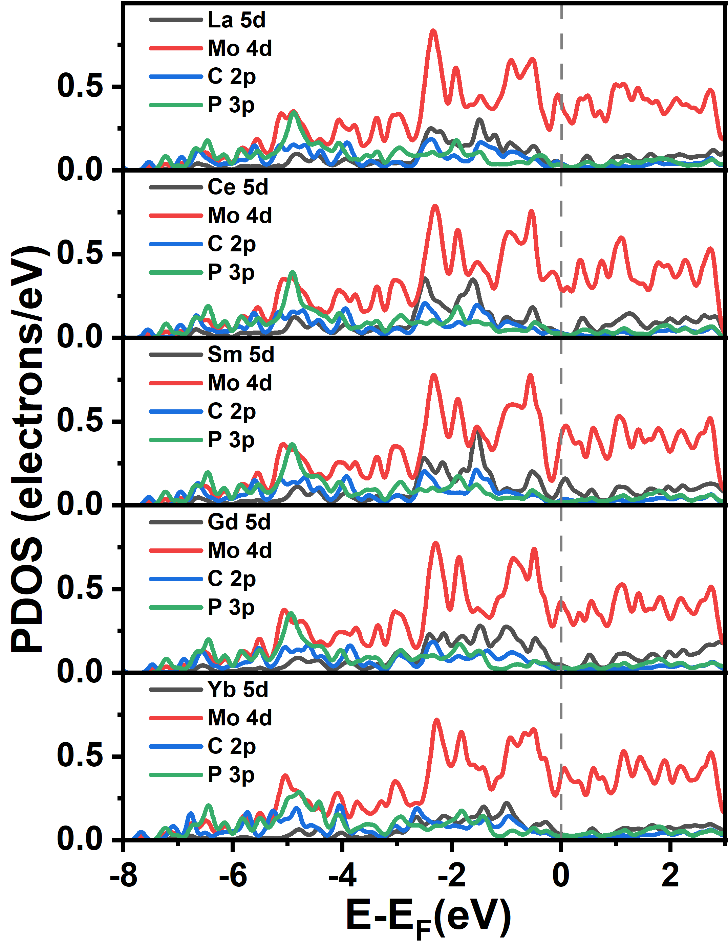


**Figure S2** PDOS of La-MoP@C, Ce-MoP@C, Sm-MoP@C, Gd-MoP@C, and Yb-MoP@C.


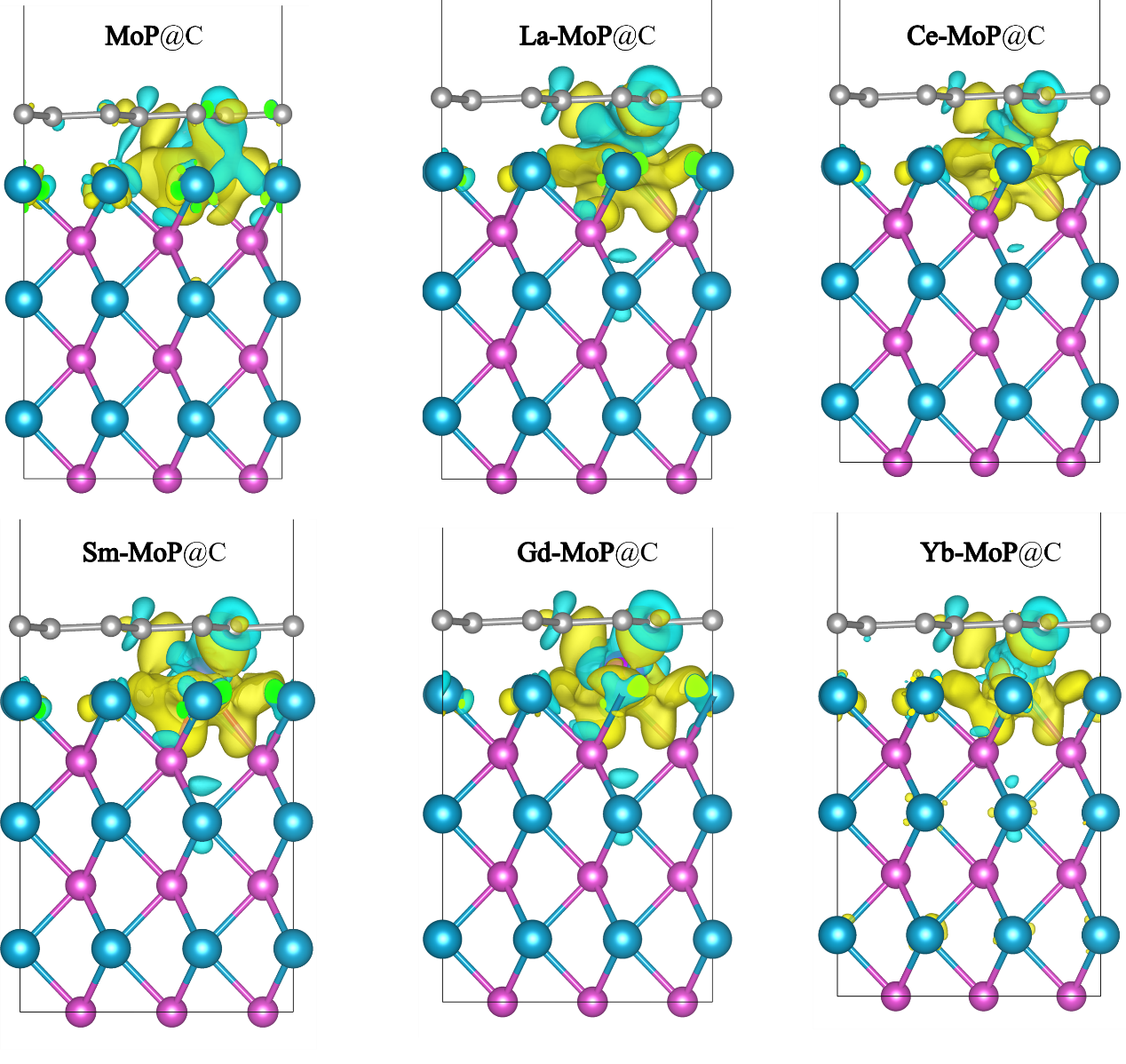


**Figure S3** The differential charge density diagrams of Ln-MoP@C.


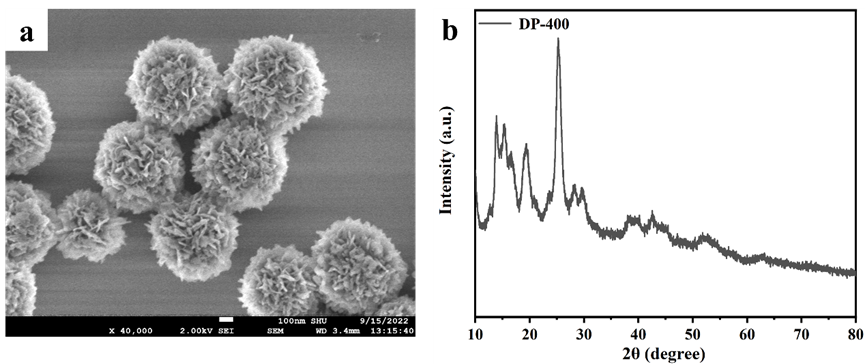


**Figure S4** SEM image and XRD pattern of DP-400.


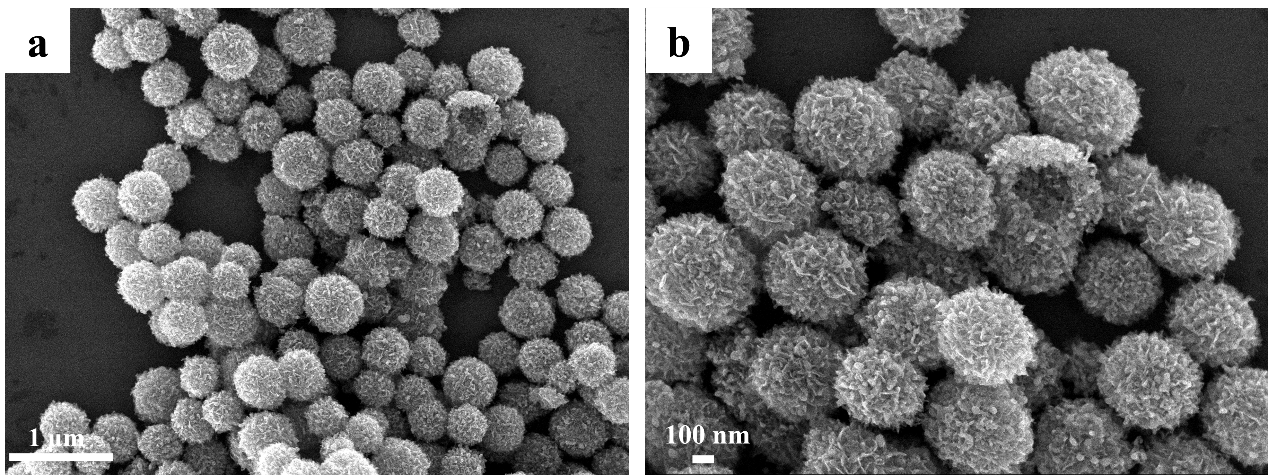


**Figure S5** SEM images of Gd-Mo@C.


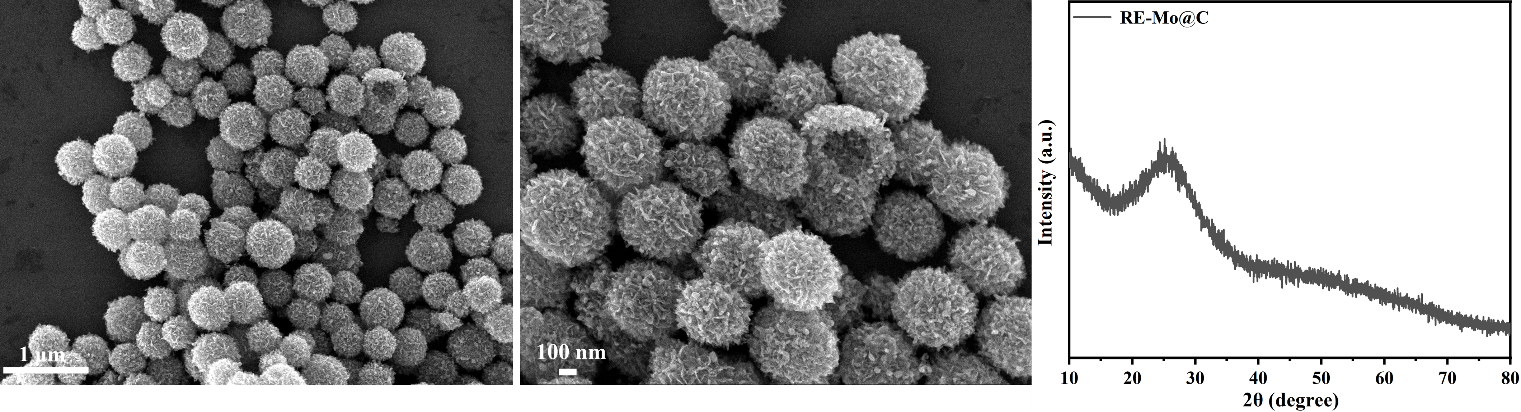


**Figure S6** XRD pattern of Gd-Mo@C.


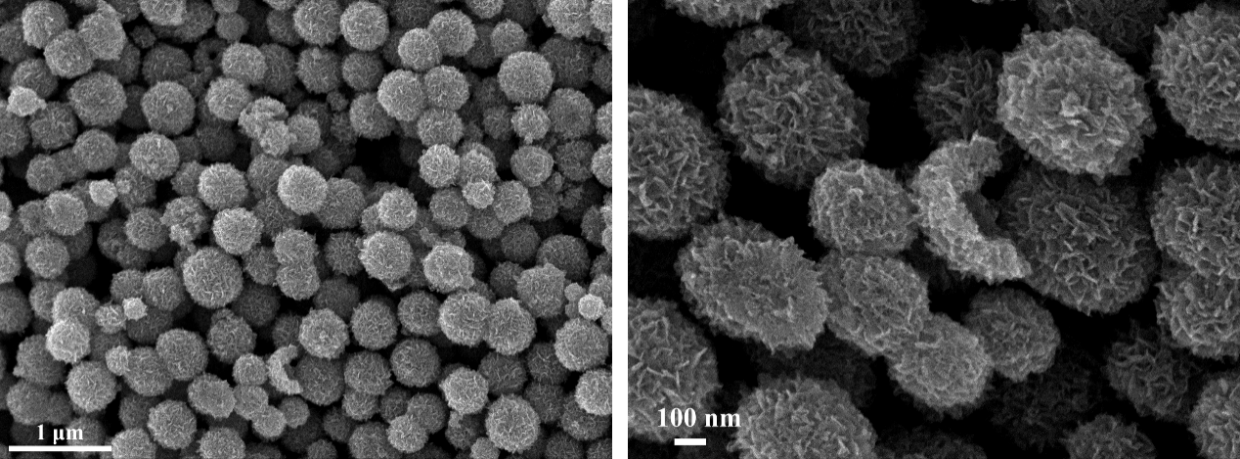


**Figure S7** SEM images of Gd-MoP@C


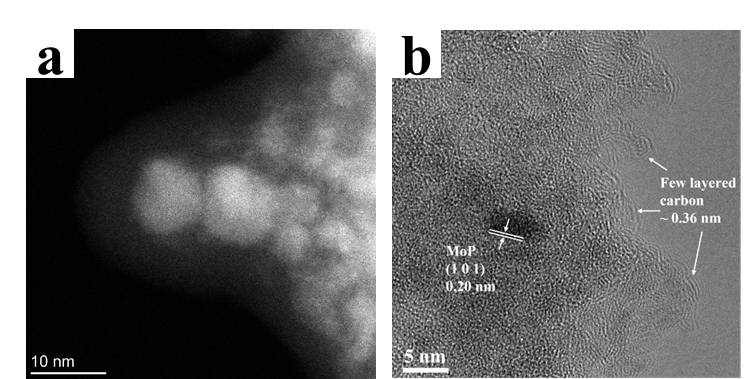


**Figure S****8** (a) HAADF–STEM image and (b) high-resolution TEM image of Gd-MoP@C.


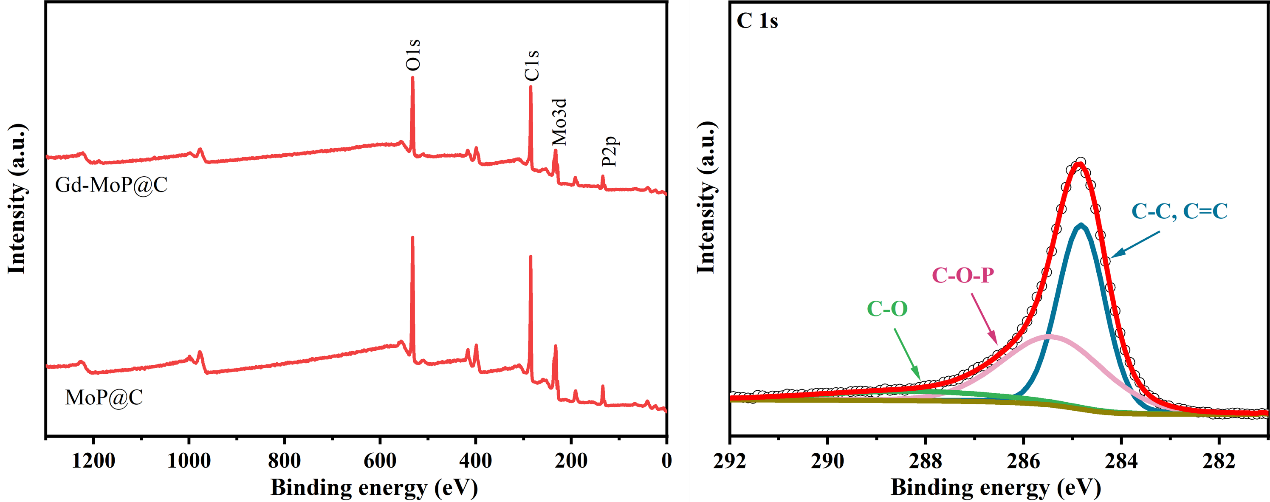


**Figure S9** XPS spectra of Gd-MoP@C and MoP@C.


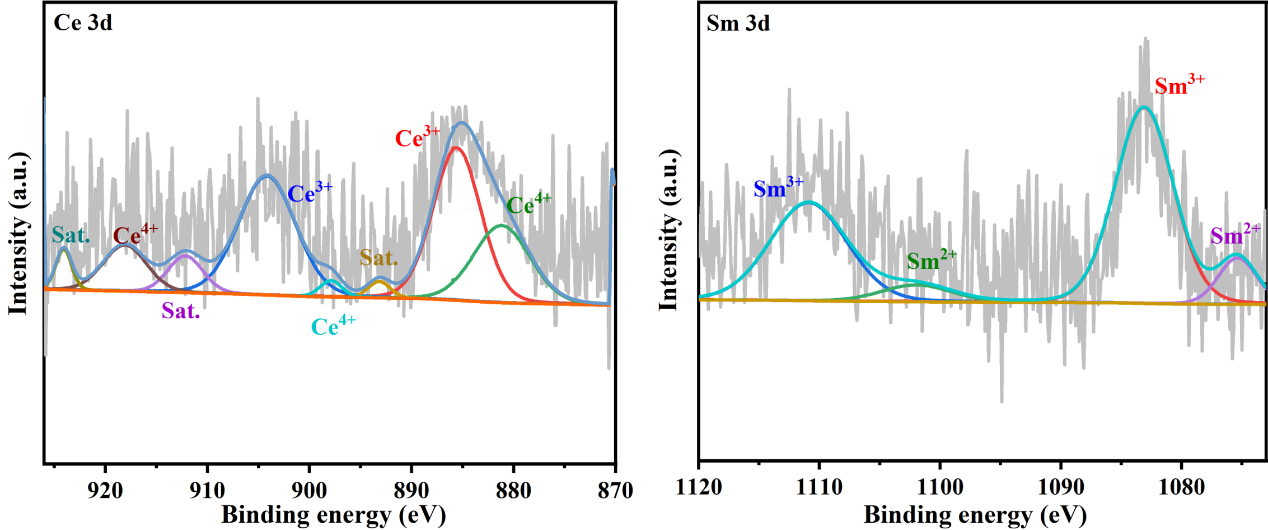


**Figure S10** High-resolution XPS spectra of Ce 3d and Sm 3d for Ce-MoP@C and Sm-MoP@C.


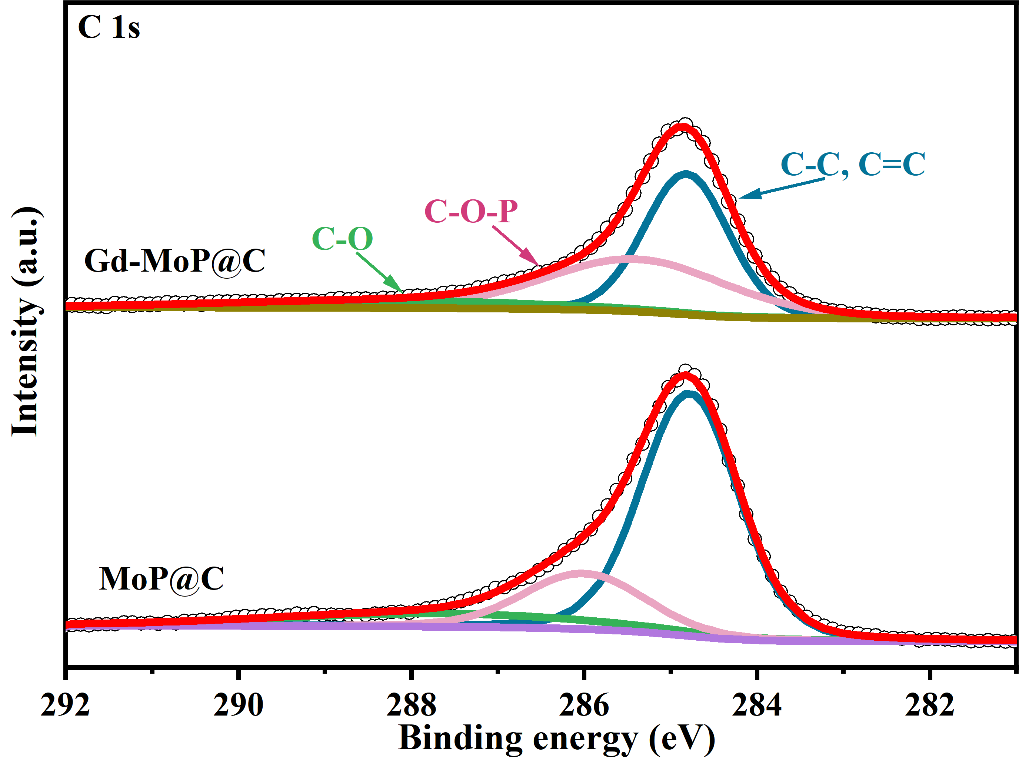


**Figure S11** High-resolution XPS spectra of C 1s for Gd-MoP@C and MoP@C.


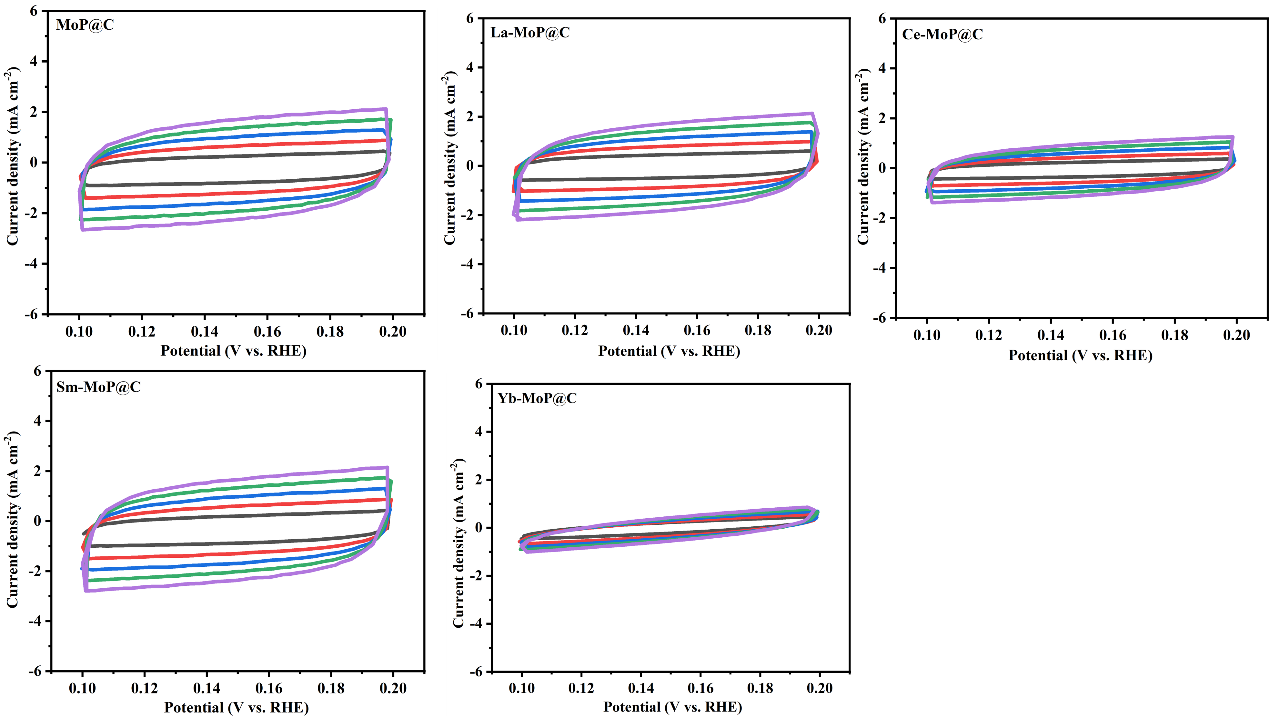


**Figure S12** ECSA of MoP@C, La-MoP@C, Ce-MoP@C, Sm-MoP@C, and Yb-MoP@C in 1 M KOH.


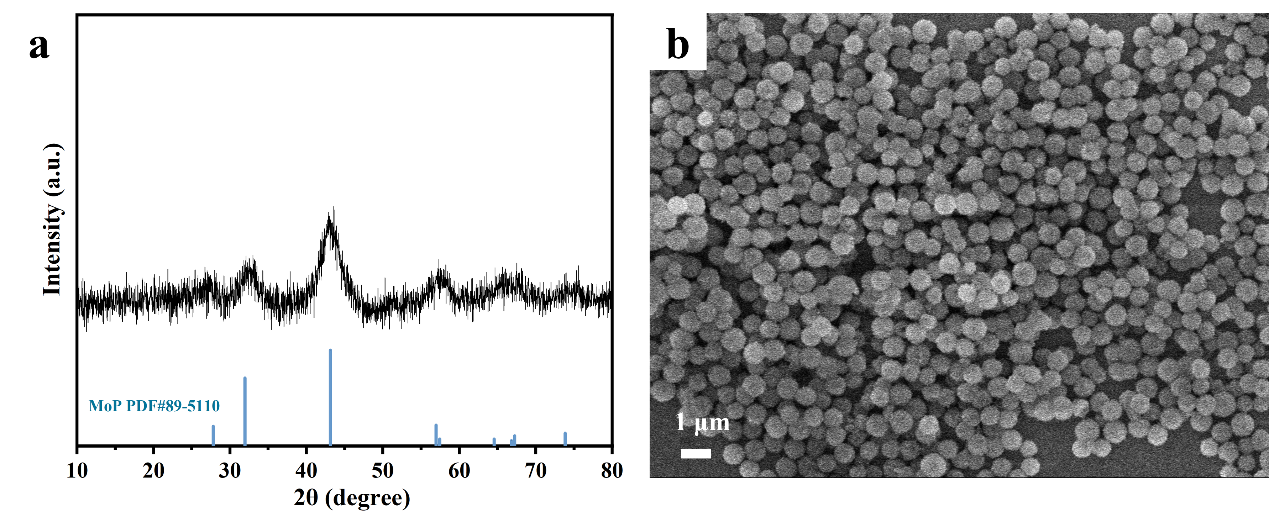


**Figure S13** XRD pattern and SEM image of Gd-MoP@C after stable test in 1 M KOH.


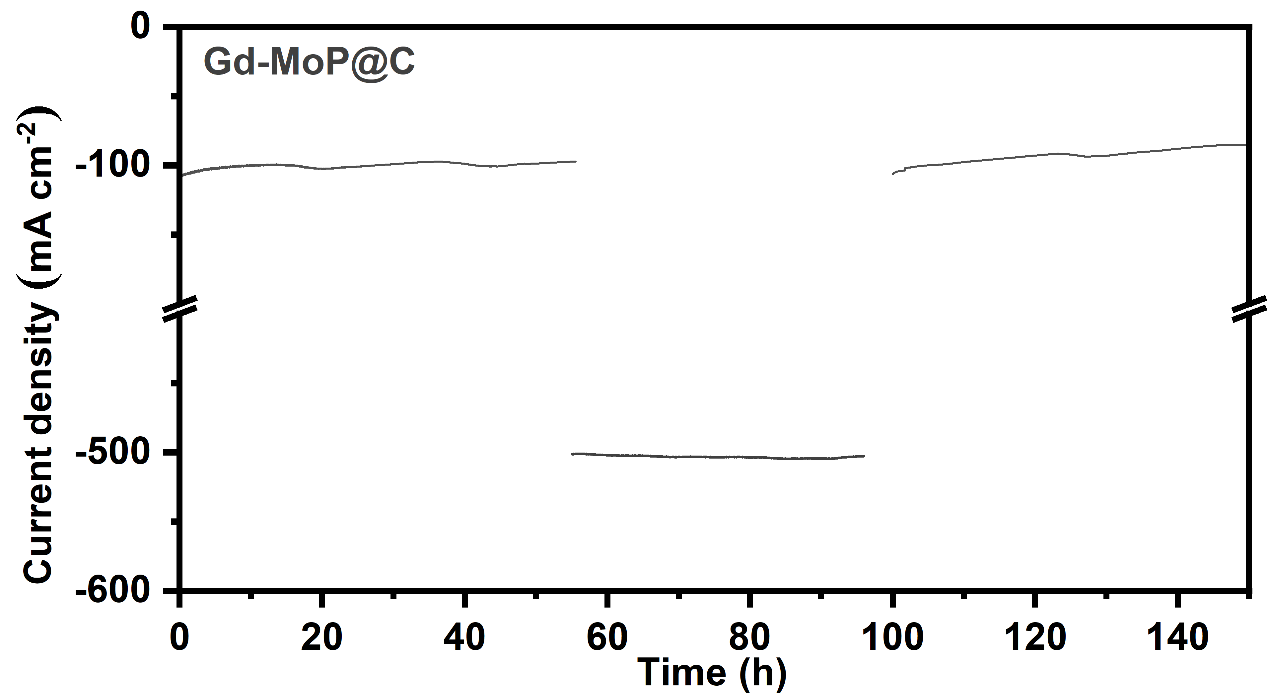


**Figure S14** I-t test for Gd-MoP@C at 100 mA cm^-2^ and 500 mA cm^-2^ in 1M KOH.


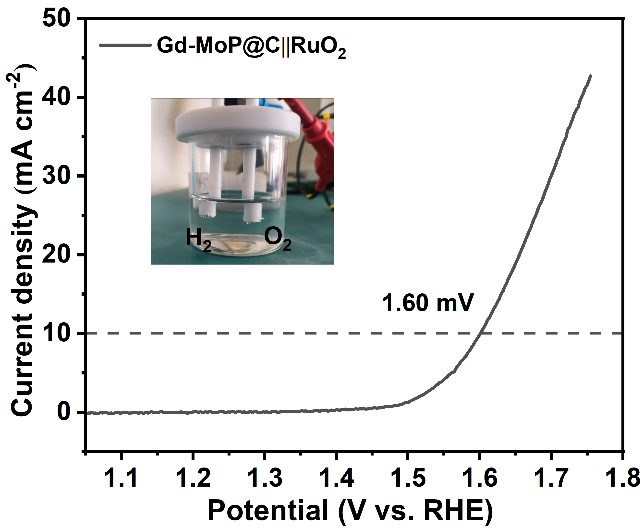


**Figure S15** Polarization curve of overall water splitting at a scan rate of 15 mV s^−1^ in 1 M KOH solution.


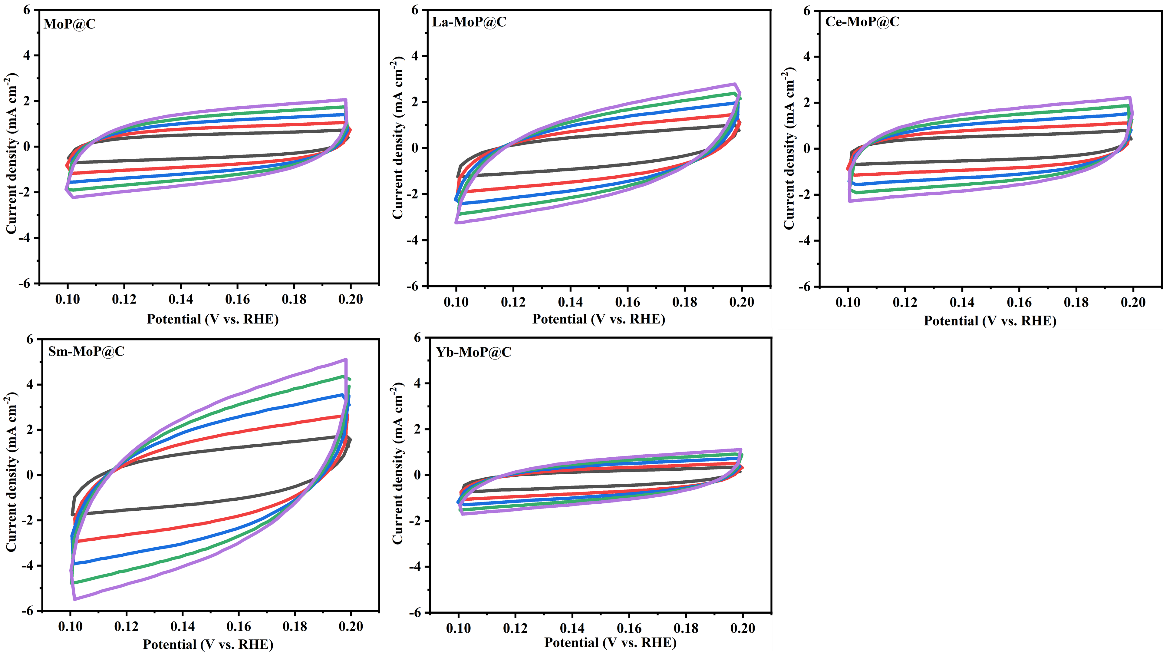


**Figure S16** ECSA of MoP@C, La-MoP@C, Ce-MoP@C, Sm-MoP@C, and Yb-MoP@C in 0.5 M H_2_SO_4_.


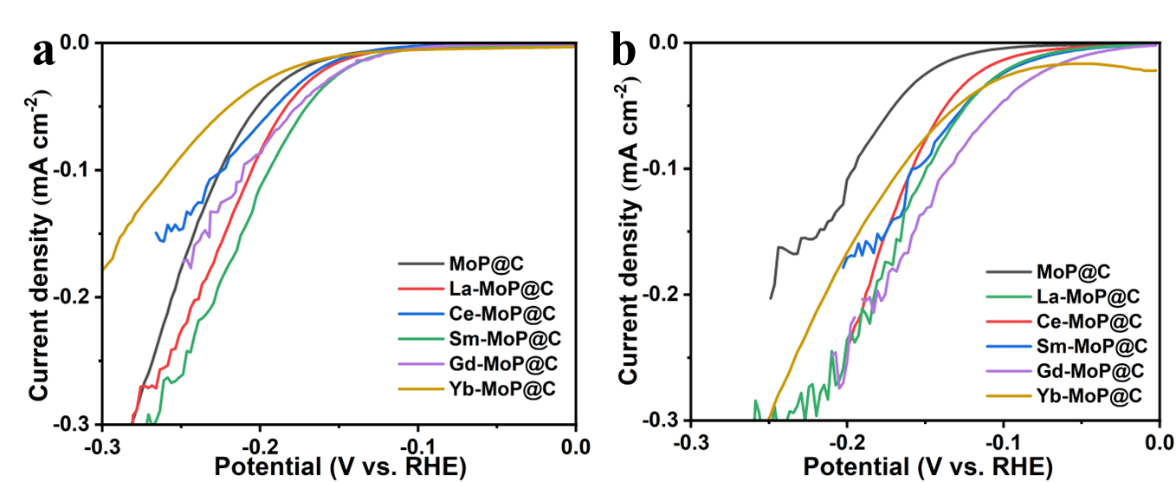


**Figure S17** The current densities of Ln-MoP@C normalized by ECSA in a) 1 M KOH and b) 0.5 M H_2_SO_4_.


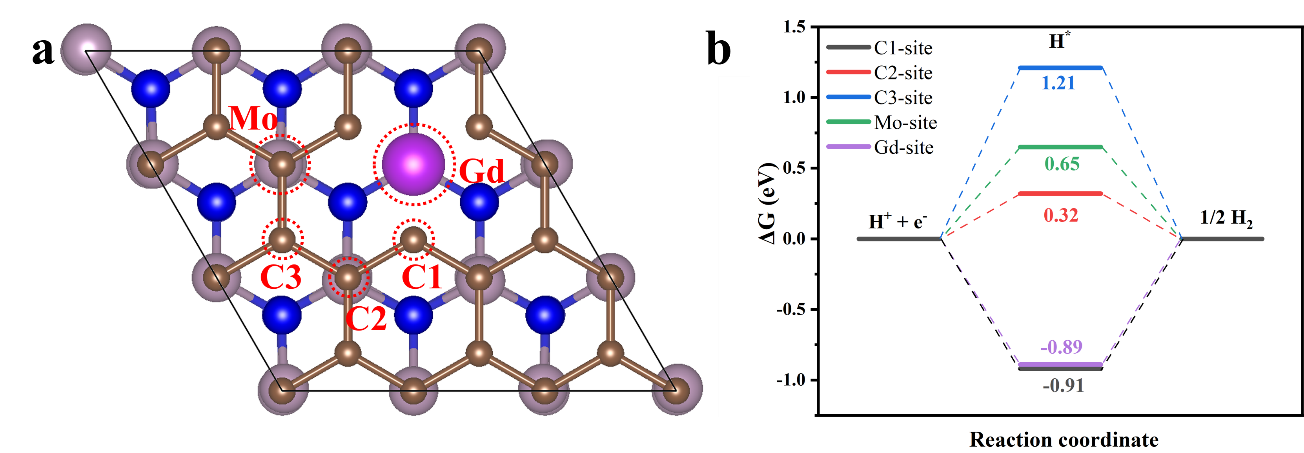


**Figure S18** a) H adsorbed sites of Gd-MoP@C structure; b) HER free energy diagrams for various sites of Gd-MoP@C.


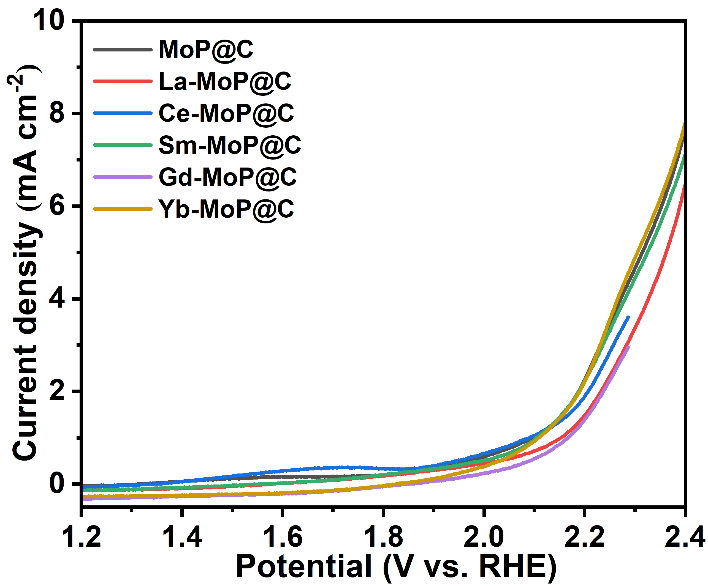


**Figure S19** LSV of Ln-MoP@C and MoP@C for OER in 1 M KOH.

“We find no significant OER activity in our Ln-MoP@Cs, which may be due to the carbon layer on the surface hindering the reconstruction of the surface groups of MoP.”

**Table S1** The Bader charge of selected atoms of Ln-MoP@C

|  | MoP@C | La-MoP@C | Ce-MoP@C | Sm-MoP@C | Gd-MoP@C | Yb-MoP@C |
| --- | --- | --- | --- | --- | --- | --- |
| RE |  | -1.27 | -1.12 | -1.25 | -1.40 | -1.31 |
| Mo | -0.66 | -0.65 | -0.43 | -0.64 | -0.64 | -0.68 |
| P | 0.52 | 0.53 | 0.35 | 0.53 | 0.53 | 0.67 |
| C | 0.20 | 0.21 | 0.17 | 0.21 | 0.22 | 0.20 |

**Table S2** Summary of the exchange current density of LnMoP@C and MoP@C.

| Catalysts | Exchange current density (mA cm^-2^) | |
| --- | --- | --- |
|  | 1 M KOH | 0.5 M H_2_SO_4_ |
| MoP@C | 1.63 | 1.52 |
| La-MoP@C | 1.76 | 1.68 |
| Ce-MoP@C | 1.24 | 1.67 |
| Sm-MoP@C | 1.99 | 1.7 |
| Gd-MoP@C | 3.02 | 1.8 |
| Yb-MoP@C | 1.01 | 1.63 |

**Table S3** Comparison of overpotentials (10 mA cm^-2^) and Tafel slopes of Gd-MoP@C catalyst and previously reported excellent MoP based HER catalysts in alkaline solutions.

| Materials | Overpotential  (η_10_, mA cm^-2^) | Tafel slope  (mV dec^-2^) | Reference |
| --- | --- | --- | --- |
| Gd-MoP@C | 74 | 62.32 | This work |
| S-MoP NPL | 104 | 56 | 1 |
| Yb-MoP@NC | 136.5 | 70 | 2 |
| La-MoP@NC | 129.3 | 58 | 2 |
| MoP@NC | 149 | 62 | 3 |
| MoP@NCHSs-900 | 92 | 62 | 4 |
| MoP-P_v_ | 151 | 71 | 5 |
| MoP@NC | 96 | 96 | 6 |
| Ni–MoP | 162 | 103 | 7 |
| MoP-Mo_2_C/NPC | 120 | 50 | 8 |
| NM/rGO-2:1 | 122 | 71 | 9 |
| Ce-doped CoMoP/MoP@C | 188 | 76 | 10 |
| La-MoP@N/C | 113 | 59.7 | 11 |
| CeNiFe-MOF | 113 | 71.3 | 12 |

**Table S4** Comparison of overpotentials (10 mA cm^-2^) and Tafel slopes of Gd-MoP@C catalyst and previously reported excellent MoP based HER catalysts in acid solutions.

| Materials | Overpotential  (η_10_, mA cm^-2^) | Tafel slope  (mV dec^-2^) | Reference |
| --- | --- | --- | --- |
| Gd-MoP@C | 134 | 57.33 | This work |
| La-MoP@NC | 142 | 58 | 2 |
| Yb-MoP@NC | 148 | 68 | 2 |
| MoP@NC | 96 | 49 | 3 |
| 0.05Mn-MoP NSs | 199 | 49 | 13 |
| Ni2P/MoP–CC | 290 | 63 | 14 |
| MoS2/MoP/NC | 151 | 58 | 15 |
| MoP@NPC-H | 141 | 59 | 16 |
| 0.05Co-MoP | 149 | 62.1 | 17 |

**References**

1. K. Liang, S. Pakhira, Z. Yang, A. Nijamudheen, L. Ju, M. Wang, C. I. Aguirre-Velez, G. E. Sterbinsky, Y. Du, Z. Feng, J. L. Mendoza-Cortes, Y. Yang, S-Doped MoP Nanoporous Layer Toward High-Efficiency Hydrogen Evolution in pH-Universal Electrolyte. *ACS Catal.* **9**, 651-659 (2019).

2. P. Wei, X. Li, Z. He, Z. Li, X. Zhang, X. Sun, Q. Li, H. Yang, J. Han, Y. Huang, Electron density modulation of MoP by rare earth metal as highly efficient electrocatalysts for pH-universal hydrogen evolution reaction. *Appl. Catal. B* **299**, 120657 (2021).

3. C. Pi, C. Huang, Y. Yang, H. Song, X. Zhang, Y. Zheng, B. Gao, J. Fu, P. K. Chu, K. Huo, In situ formation of N-doped carbon-coated porous MoP nanowires: a highly efficient electrocatalyst for hydrogen evolution reaction in a wide pH range. *Appl. Catal. B* **263**, 118358 (2020).

4. D. Zhao, K. Sun, W.-C. Cheong, L. Zheng, C. Zhang, S. Liu, X. Cao, K. Wu, Y. Pan, Z. Zhuang, B. Hu, D. Wang, Q. Peng, C. Chen, Y. Li, Synergistically Interactive Pyridinic-N–MoP Sites: Identified Active Centers for Enhanced Hydrogen Evolution in Alkaline Solution. *Angew. Chem. Int. Ed.* **59**, 8982-8990 (2020).

5. H. Ma, W. Yan, Y. Yu, L. Deng, Z. Hong, L. Song, L. Li, Phosphorus vacancies improve the hydrogen evolution of MoP electrocatalysts. *Nanoscale* **15**, 1357-1364 (2023).

6. J. Li, H. Huang, X. Cao, H.-H. Wu, K. Pan, Q. Zhang, N. Wu, X. Liu, Template-free fabrication of MoP nanoparticles encapsulated in N-doped hollow carbon spheres for efficient alkaline hydrogen evolution. *Chem. Eng. J* **416**, 127677 (2021).

7. W. Xiao, L. Zhang, D. Bukhvalov, Z. Chen, Z. Zou, L. Shang, X. Yang, D. Yan, F. Han, T. Zhang, Hierarchical ultrathin carbon encapsulating transition metal doped MoP electrocatalysts for efficient and pH-universal hydrogen evolution reaction. *Nano Energy* **70**, 104445 (2020).

8. E. Jiang, J. Li, X. Li, A. Ali, G. Wang, S. Ma, P. Kang Shen, J. Zhu, MoP-Mo_2_C quantum dot heterostructures uniformly hosted on a heteroatom-doped 3D porous carbon sheet network as an efficient bifunctional electrocatalyst for overall water splitting. *Chem. Eng. J* **431**, 133719 (2022).

9. L. Zhang, W. Xiao, Y. Zhang, F. Han, X. Yang, Nanocarbon encapsulating Ni-doped MoP/graphene composites for highly improved electrocatalytic hydrogen evolution reaction. *Composites Communications* **26**, 100792 (2021).

10. T. Chen, Y. Fu, W. Liao, Y. Zhang, M. Qian, H. Dai, X. Tong, Q. Yang, Fabrication of Cerium-Doped CoMoP/MoP@C Heterogeneous Nanorods with High Performance for Overall Water Splitting. *Energy & Fuels* **35**, 14169-14176 (2021).

11. X. Ye, H. Ma, S. Wu, F. Wu, X. Zhuge, J. Liu, Y. Ren, P. Wei, Electron structure customization of molybdenum phosphide via lanthanum doping toward highly efficient overall water splitting, *J Mater Sci Technol* **218**, 227 (2025).

12. J. Yang, Y. Shen, J. Xian, R. Xiang, G. Li, Rare-earth element doped NiFe-MOFs as efficient and robust bifunctional electrocatalysts for both alkaline freshwater and seawater splitting, *Chem. Sci* **16**, 685-692 (2025)

13. Z. Mu, T. Guo, H. Fei, Y. Mao, Z. Wu, D. Wang, Mn-doped porous interconnected MoP nanosheets for enhanced hydrogen evolution. *Appl. Surf. Sci.* **551**, 149321 (2021).

14. Y. Xu, M. Yan, Z. Liu, J. Wang, Z. Zhai, B. Ren, X. Dong, J. Miao, Z. Liu, Nanostructures Ni_2_P/MoP@N – doping porous carbon for efficient hydrogen evolution over a broad pH range. *Electrochim. Acta* **363**, 137151 (2020).

15. J.-Q. Chi, Y.-M. Chai, X. Shang, B. Dong, C.-G. Liu, W. Zhang, Z. Jin, Heterointerface engineering of trilayer-shelled ultrathin MoS_2_/MoP/N-doped carbon hollow nanobubbles for efficient hydrogen evolution. *J. Mater. Chem. A* **6**, 24783-24792 (2018).

16. J.-Q. Chi, W.-K. Gao, L.-M. Zhang, B. Dong, K.-L. Yan, J.-H. Lin, B. Liu, Y.-M. Chai, C.-G. Liu, Induced phosphorization-derived well-dispersed molybdenum phosphide nanoparticles encapsulated in hollow N-doped carbon nanospheres for efficient hydrogen evolution. *ACS Sustainable Chemistry Engineering* **6**, 7676-7686 (2018).

17. Y. Liu, C. Yue, F. Sun, W. Bao, L. Chen, Z. Zeb, C. Wang, S. Ma, C. Zhang, D. Sun, Y. Pan, Y. Huang, Y. Lu, Y. Wei, Superhydrophilic molybdenum phosphide quantum dots on porous carbon matrix for boosting hydrogen evolution reaction. *Chem. Eng. J* **454**, 140105 (2023).
